# A Stepwise Assessment of Parsimony and Fuzzy Entropy in Species Distribution Modelling

**DOI:** 10.3390/e23081014

**Published:** 2021-08-05

**Authors:** Alba Estrada, Raimundo Real

**Affiliations:** Biogeography, Diversity and Conservation Research Team, Department of Animal Biology, Universidad de Málaga (UMA), 29010 Málaga, Spain

**Keywords:** Akaike Information Criterion, favourability function, fuzzy entropy, probability of occurrence, Shannon entropy, species distribution models, stepwise logistic regression, uncertainty

## Abstract

Entropy is intrinsic to the geographical distribution of a biological species. A species distribution with higher entropy involves more uncertainty, i.e., is more gradually constrained by the environment. Species distribution modelling tries to yield models with low uncertainty but normally has to reduce uncertainty by increasing their complexity, which is detrimental for another desirable property of the models, parsimony. By modelling the distribution of 18 vertebrate species in mainland Spain, we show that entropy may be computed along the forward-backwards stepwise selection of variables in Logistic Regression Models to check whether uncertainty is reduced at each step. In general, a reduction of entropy was produced asymptotically at each step of the model. This asymptote could be used to distinguish the entropy attributable to the species distribution from that attributable to model misspecification. We discussed the use of fuzzy entropy for this end because it produces results that are commensurable between species and study areas. Using a stepwise approach and fuzzy entropy may be helpful to counterbalance the uncertainty and the complexity of the models. The model yielded at the step with the lowest fuzzy entropy combines the reduction of uncertainty with parsimony, which results in high efficiency.

## 1. Introduction

Species distribution models are widely employed by the scientific community to detect species’ suitable areas in a territory [1,2], to understand the environmental variables that define species distributions [3,4], to forecast the effect of global changes on their distributions [5,6], to detect priority areas for conservation [7,8], or to guide conservation programmes [1,9]. There are a wide variety of procedures to perform species distribution models, including Generalized Linear Models [10], Generalized Additive Models [11], Artificial Neural Networks [12], Maximum Entropy Models (MaxEnt) [13], and Classification and Regression Trees [14].

Some of these approaches, such as MaxEnt, are based on the concept of entropy [15]. Shannon [16] defined entropy in the context of the information theory (see formulation in Methods) as a measure of information, choice, and uncertainty. It reflects the uncertainty associated with a probability distribution, i.e., a broad distribution represents more uncertainty than does a sharply peaked one [17], and represents the degree of disorganisation of a system [18]. Although the concept of entropy can be applied to different systems, it is an intrinsic characteristic of the geographic distribution of a species, as a distribution with higher entropy involves more uncertainty or choices [13], i.e., it is more gradually constrained by the environmental conditions, whereas a distribution with lower entropy is more abruptly constrained by the environment. With maximum entropy, the distribution of the species is completely disordered, i.e., the probability of occurrence is equally distributed in the entire territory. The smaller the entropy, the more orderly the distribution of the species is, i.e., more clearly suitable and unsuitable areas are distinguished. Therefore, performing the models taking into account the concept of entropy is justified. MaxEnt estimates target species suitability by finding the probability distribution of maximum entropy (i.e., that is most spread out, or closest to uniform), subject to a set of variables that represent our incomplete information about the target species distribution [13]. Model selection using information theory is also based on the entropy principle because selecting the model with the lowest Akaike Information Criterion (AIC) is equivalent to minimising the entropy [19]. Stepwise Generalised Linear Models, and more specifically stepwise logistic regression, a commonly used supervised machine learning algorithm [20,21], can also be considered to be related to the concept of entropy. In this case, the goal is to find the model that gives the maximum reduction of entropy in each of the steps. This can be computed with the AIC of the steps but also with Shannon entropy and fuzzy entropy (see below).

In stepwise modelling, each step of inclusion or deletion of variables yields a model, and predictions can be obtained from each of the steps. This can be useful to assess if a model with just the first few variables could already provide predictions close to the final step, or to see which variables determine the more general trends in the model predictions (i.e., those variables that first become part of the model), and which of them act on finer-scale distribution patterns (i.e., those entering last) [1]. We can also calculate the entropy of the final model and the entropy of those produced at each step. This can be done by applying Shannon entropy to the probabilities obtained in each of the steps. Additionally, there is also a definition of fuzzy entropy (see formulation in Methods) that can be applied to fuzzy sets [22]. Related to fuzzy sets, favourability functions [23] can be used to identify favourable areas for the occurrence of a species in a territory. The favourability value for a species in a cell can be interpreted as the degree of membership of the cell to the fuzzy set of cells that are favourable for the species [24]. Therefore, fuzzy operations, such as fuzzy entropy, can be applied to the favourability values. Fuzzy entropy also reflects the uncertainty of a system.

In this paper, we showed that forward-backwards stepwise selection of variables in logistic regression generally increases the complexity of the models while reducing their uncertainty, as the entropy tends to be reduced asymptotically at each step with respect to the previous step. This characteristic of the procedure may be used to counterbalance the complementary properties of parsimony and certainty, and to distinguish the entropy due to disorganisation in the species distribution from that, due to deficiency in the model. We also discussed the differential suitability of Shannon entropy, the AIC, and fuzzy entropy for this purpose.

## 2. Materials and Methods

### 2.1. Modelling Method

We modelled the distribution of 18 vertebrate species in mainland Spain (Table 1). Species included taxa of the four terrestrial vertebrate groups, i.e., amphibians, reptiles, mammals, and birds. We chose only species whose distributions are restricted or mainly restricted to the Iberian Peninsula. In the case of birds, distribution referred to breeding sites. Presence/absence in each 10 km × 10 km Universal Transverse Mercator cell was considered according to the distribution atlases of vertebrates in Spain [25,26,27]. Although each of the atlases has its own specifications, in general, a cell was considered a presence for a species when an individual was recorded in the cell; otherwise, the cell was considered an absence for the species.

To model the distribution of each species, we considered a pool of environmental variables related to four predictor sets, i.e., climate, topography, lithology, and human activity (Table S1 in Supplementary Materials). We performed a logistic regression of the distribution of each species on each environmental variable and checked the significance of these univariate predictive models according to Rao’s score test. The values of the parameters of the logistic regression were established by maximum likelihood estimation using a gradient ascent machine learning algorithm.

The number of variables, especially within the climate predictor set, was large and many of them showed high values of correlation. We performed a species-specific selection of variables in three stages. First, for each species and predictor set, we calculated pairwise correlations between the variables and, among those pairs of variables with a Pearson correlation value above 0.8, we excluded the one with the least significant individual relationship with the distribution of the species. In this way, among variables correlated and belonging to the same predictor set, which provides similar information, we chose the most informative one for each species. Second, with the variables selected in the first step, we calculated the false discovery rate to control for the increase in Type I error caused by the number of remaining predictor variables under a false discovery rate value of q = 0.05 [28,29,30]. We only retained in the analysis those variables that were significantly (*p* < 0.05) related to the distribution of the species in univariate models under a false discovery rate of q < 0.05 (i.e., given the overall pool of predictor variables evaluated, the probability that a non-informative variable is retained for the subsequent procedures is lower than 0.05). This procedure orders the variables according to decreasing significance (increasing p-value), being I the position of each variable in this ordered list. Then, only variables up to the highest I position whose p-value is lower than I*(q/V) were accepted, where V is the total number of variables selected in the first step. Finally, we performed a multivariate forward-backwards stepwise logistic regression based on the AIC on the variables that were retained in the FDR test.

Logistic regression follows the expression:

$$P=\frac{e^{y}}{1+e^{y}}$$

where *P* is the probability of the presence of the species, *e* is the basis of the natural logarithm, and *y* is a logit equation of the form:

$$y=\alpha +\beta_{1}x_{1}+\beta_{2}x_{2}+\ldots +\beta_{n}x_{n}$$

where α is the intercept and $\beta_{1},\;\beta_{2},\ldots ,\;\beta_{n}$ are the coefficients of the *n* environmental predictors $x_{1},\;x_{2},\ldots ,\;x_{n}$ included into the model.

In a forward-backwards stepwise multivariate model, the model starts with the intercept or null model, and at each following step, the model alternates between forward selection and backward elimination [18]. After each step of forward inclusion, all the variables in the model are tested, and those that do not fulfil the AIC criterion are excluded before the next forward selection step [31]. We used the AIC because we were interested in the predictive capacity of the models, as Akaike derived the AIC from a predictive perspective [32]. The values of the parameters α and *β_i_* in the logit equation were established by maximum likelihood estimation using a gradient ascent machine learning algorithm.

Thus, the output of the logistic regression model is a probability value. Probability depends both on the response of the species to the predictors and on the overall prevalence of the species [33] (prevalence being the ratio between presences and the total number of cells). To remove the effect of prevalence from the model output in order to make results comparable among species, we applied the favourability function proposed by Real, Barbosa, and Vargas [23]:

$$F=\left[P/\left(1-P\right)]/[\left(n_{1}/n_{0}\right)+\left(P/(1-P\right)\right)]$$

where *P* is the probability value in a cell, *n*_1_ is the total number of presences, and *n*_0_ is the total number of absences in the dataset.

The favourability function reflects the degree (between 0 and 1) to which the local probability values differ from that expected according to the species prevalence, where *F* = 0.5 corresponds to *P* = prevalence. Favourability values only reflect the response of the species to the predictors [24] and (unlike probability) can be regarded as the degree of membership of the localities to the fuzzy set of sites with conditions that are favourable for the species [24], which enables the easy application of fuzzy logic operations to distribution modelling [34]. Fuzzy logic operations expand the potential of the favourability function for comparison between models [7,35,36]. Thus, it is possible to calculate the fuzzy entropy (see below).

We assessed the classification power of the models, i.e., how well presences and absences are classified as such, by calculating Cohen’s kappa (correct classification over chance), sensitivity (correct prediction of presences), specificity (correct prediction of absences), and their Correct Classification Rate (overall accuracy), using the favourability value of *F* = 0.5 as classification threshold. We evaluated the discrimination capacity using the Area Under the Curve (AUC) of the Receiver Operating Characteristic, which is independent of any favourability threshold. The goodness-of-fit of the models was assessed by showing the calibration plot (observed prevalence against predicted probability) and using the Hosmer and Lemeshow test for 10 bins of probabilities (each one with a range of 0.1) [37] (obtaining a p-value of difference between observations and predictions). The compensation between goodness-of-fit and complexity of the models was assessed using the AIC.

All analyses were performed in R [38] with the packages *fuzzySim* [39,40] and *modEvA* [41]. Specifically, we used the functions *corSelect*, *multGLM* and *stepByStep* of *fuzzySim*, and *multModEv* of *modEvA*. We produced maps of the predictions with the package *maptools* [42] and represented them in QGIS [43].

### 2.2. Entropy Calculations

The formulation of entropy (*H*) developed by Shannon in 1948 [16] was as follows:

$$H=-\overset{n}{\underset{i=1}{\sum }}P_{i}\log P_{i}$$

where *P_i_* are the set of probabilities, with ∑*P_i_* = 1; log is the natural logarithm; and *n* is the sample size.

One property of *H* is that, for a given *n*, *H* is a maximum and equal to *log (n)* when all the *P_i_* are equal (i.e., 1*/n*) [16]. In our case, *n* is the total number of cells, i.e., 5308, and to satisfy the above-mentioned property, all probabilities must sum to 1 [13]. This makes sense when analysing the probability that a unique event occurs in a finite universe (for example, the probability that a sampled individual belongs to each of many sampled species), which in our case would imply that there is only a presence in the set of cells. If the species has more than one presence in the study area, the sum of probabilities in all the cells is equal to the number of presences. To satisfy the condition that ∑*P_i_* = 1, we divided the probability of presence of the species in each of the cells by the number of presences of the species and then applied the formula of the Shannon entropy. Notice that this has a mathematical justification but not a biogeographical sense (see Discussion). For each species, we calculated the Shannon entropy at each of the steps of the stepwise procedure.

We also calculated a fuzzy entropy (*R*) with the favourability values. The fuzzy entropy is defined as [22]:

$$R=\frac{\sum_{i=1}^{n}\left(F_{i}\cap F_{i}^{c}\right)}{\sum_{i=1}^{n}\left(F_{i}\cup F_{i}^{c}\right)}$$

where *F_i_* is the favourability value in a cell *i*; *n* is the total number of cells; $F_{i}^{c}$ is the complement of *F_i_* ($F_{i}^{c}$ = 1 − *F_i_*); ∩ is the fuzzy intersection, i.e., the minimum value between the favourability and its complement; and U is the fuzzy union, i.e., the maximum value between the favourability and its complement [44].

Note that we did not transform favourability values to obtain the fuzzy entropy. Fuzzy entropy has values between zero and one. If fuzzy entropy is one, the distribution of the species is completely disordered, i.e., favourability is equally distributed in the entire territory with *F_i_* = 0.5. The smaller the entropy, the more orderly the distribution of the species is, i.e., the model more clearly distinguished between presences and absences. For each species, we also calculated the fuzzy entropy at each of the steps of the stepwise procedure.

## 3. Results

Although the initial candidate variables were 86, the number of variables selected after controlling the pairwise correlations was between 20 and 24, the number selected after performing the FDR test was between 15 and 22, and finally, the number of variables that formed part of the models of the different species, i.e., those selected in the stepwise approach, was between 6 and 18 (see Supplementary Materials S1). Models included variables of the four predictor sets considered (climate, topography, lithology, and human activity) for all the species except four of them that included variables of three predictor sets. Final models had good discrimination and classification capacity and goodness-of-fit [average and range values for the 18 species: AUC: 0.929 (0.781–0.999), kappa: 0.358 (0.118–0.549), sensitivity: 0.905 (0.736–1), specificity: 0.852 (0.646–0.989), Correct Classification Rate: 0.859 (0.683–0.989), Hosmer and Lemeshow test: 16.137 with *p*: 0.243 (2.513–72.795 with *p* 0–0.783)]. Individual models for all the species, their evaluation metrics, and the resulting maps are shown in Supplementary Materials S1.

**Figure 1:**
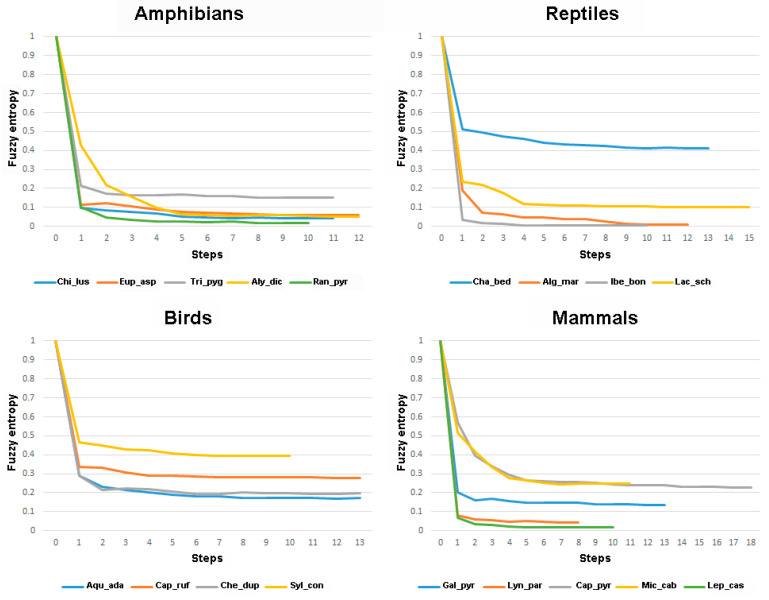
Graphs showing fuzzy entropy at each of the steps of the stepwise procedure for all analysed species, separated according to taxonomic group. Exact values are given in Supplementary Materials S1. Species codes as in Table 1.

Minimum Shannon entropy of the models for the different species ranged between 3.80 and 8.27 (maximum possible value was 8.58), and minimum fuzzy entropy ranged between 0.0057 and 0.41 (maximum possible value was 1). Most distributions had low entropy, especially when using the fuzzy entropy, which indicates that they are restricted to a certain range of the predictors. In general, both Shannon and fuzzy entropy tended to go down in each step of the model. The exceptions appeared when a variable was removed in a step or at the latest steps of the stepwise procedure but always with minor differences with the previous step, i.e., the differences appeared at the third or fourth decimal place of the entropy value. However, there were four species (*Iberolacerta bonnali*, *Chersophilus duponti*, *Microtus cabrerae*, and *Lepus castroviejoi*) for which the step with the lowest fuzzy entropy was located in the middle of the stepwise procedure (Table 1 and Supplementary Materials S1). In any case, for all analysed species, even for these four, the correlation between the favourability values at the step with the lowest fuzzy entropy and those of the final favourability model was always >0.90 (Supplementary Materials S1). This is because fuzzy entropy went down meaningfully at the first steps, and then the values reached an asymptote with similar values to the final steps (Figure 1).

**Figure 2:**
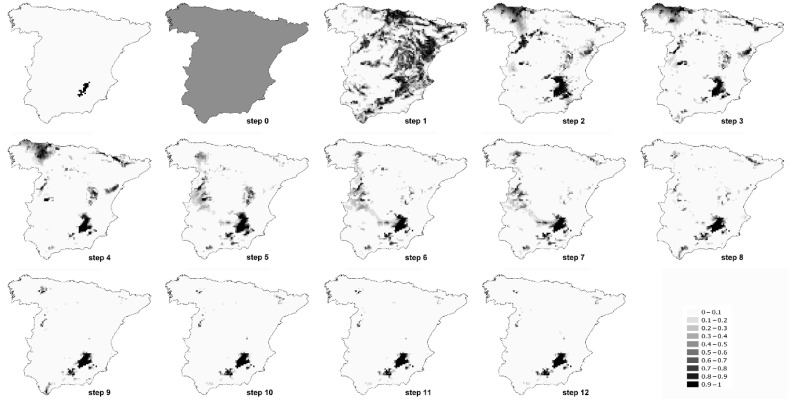
Favourability values of each step of the model of *Algyroides marchi*. The first map represents the distribution of the species in mainland Spain.

**Figure 3:**
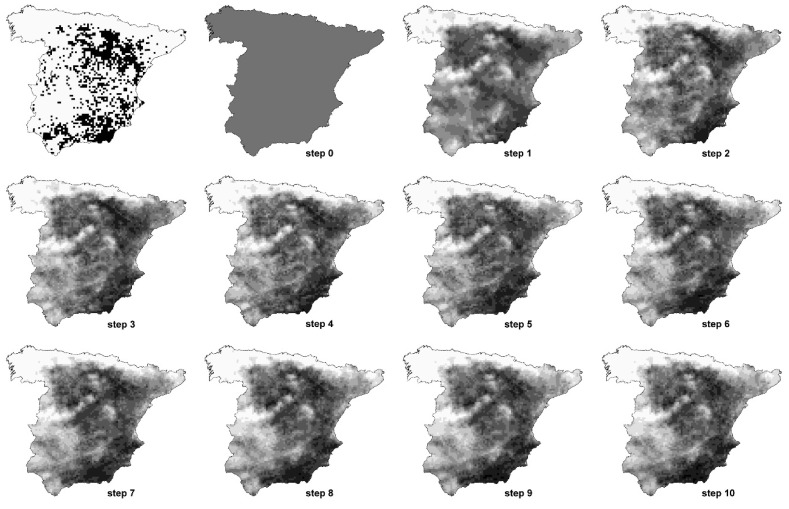
Favourability values of each step of the model of *Sylvia conspicillata*. The first map represents breeding distribution of the species in mainland Spain. Colour ramp as in Figure 1.

As examples, in Table 2 and Table 3, we show the entropy of each step of the models of two species, *Algyroides marchi* and *Sylvia conspicillata*. The entropy of each of the steps for all the species can be seen in Supplementary Materials S1. In Figure 2 and Figure 3, we represent the favourability values of each step of the models for both example species. The fuzzy entropy of step 0 is equal for all the species, and it is always equal to the unity. In step 0, the only parameter to form part of the model is the intercept, which means that the probability of presence in all the cells is equal to the prevalence of the species. Therefore, the favourability in all the cells in step 0 is equal to 0.5, whichever the prevalence of the species.

## 4. Discussion

Our approach to variable selection in three stages and with a stepwise selection in the final stage allowed us to test 86 variables that we deemed potentially relevant for the species distributions. It is common practice to include a low number of variables in the models [45,46] because many modelling techniques do not allow for the selection of variables [47], and strongly correlated variables are generally avoided beforehand [18,48]. Here, we have shown that starting the modelling procedure with 86 candidate variables results in models with a relatively low number of variables (between 6 and 18, depending on the analysed species). We controlled the correlation of the variables and the increase in type I errors in a species-specific way and performed a stepwise approach to reduce variable redundancy and increase parsimony. The inclusion of correlated variables may have an undesirable effect if the intention is to extrapolate the model to other areas or periods of time and if the pattern of the correlation is not maintained in the new environment or time scale [49]. The advantage of controlling correlation on a species-specific basis instead of selecting little correlated variables from the beginning is that different species may have the highest significant relationship with different variables, so we selected for each species those variables that have more influence on its distribution. This means that we focused our attention on the species and, therefore, the pool of variables that were included in the stepwise regression was different for the different species, which produced more realistic and biologically plausible models.

Our approach also highlighted the importance of non-climatic variables as drivers of the species distributions, although the number of climatic variables in the variable pool was disproportionately large. In species distribution models, the inclusion of climatic variables is a common practice [2,50] as it is reasonable to think that climate may have an important effect on species’ distributions, especially at broad scales [51,52]. Indeed, we found that climatic variables formed part of the models of the 18 analysed species (Supplementary Materials S1). However, we also showed that other predictors related to topography, lithology, and human activity were included in the models, and therefore they have an influence on species’ distributions. This is in accordance with studies that use more variables than only climatic ones in the modelling procedure [5,53,54]. 

In general, we obtained a good discrimination capacity of the models according to AUC. However, although this metric is commonly used to select good vs. bad models (e.g., [45]), Lobo et al. [55] already warned against using it as a general measure of model performance. Our results show that AUC is, in general, inversely related to the entropy of the species, particularly to the fuzzy entropy (Pearson correlation between AUC and fuzzy entropy and between AUC and Shannon entropy were −0.993 and −0.842, respectively, while the correlation between AUC and AIC was −0.907). More disorderly distributions (i.e., those less constrained) are more difficult to discriminate, whichever the type of model applied. The majority of our study species had AUC values ≥ 0.9, which is considered as outstanding discrimination capacity [37]; three of them had values between 0.8–0.9, and two of them had only acceptable discrimination capacity (AUC: 0.7–0.8). These last two species are *Chalcides bedriagai* and *Sylvia conspicillata*, which, in fact, are the species with the highest fuzzy entropy: 0.411 and 0.396, respectively (Supplementary Materials S1). The low AUC values of these two species do not directly mean that models for these species were worse than those produced for other species; rather, it means that the models predict wider and more gradual distributions along the available environmental domain, which is intrinsically related to higher entropy and uncertainty. AUC values measure the discrimination capacity of the models, and this is a characteristic only of the models, whereas entropy may be a characteristic of the model and of the species distribution. It is important to disentangle these two sources of entropy, i.e., whether lower values of AUC, with higher values of entropy, indicate that the intrinsic distribution of the species is more general or if the models are deficient. In the first case, lower values of AUC are perfectly expected, whereas in the second case, an improvement of the model should be pursued.

Calibration plots could be used to this end. The calibration plots of *Chalcides bedriagai* and *Sylvia conspicillata* show that the model for the latter species is very well calibrated, while the model for *Chalcides bedriagai* still needs some improvement because the model fails in the calibration of probability values higher than 0.5 (Supplementary Materials S1 and S2). The calibration plots of the models produced along the stepwise procedure for *Chalcides bedriagai* show that, although the discrimination capacity is improving and the entropy is being reduced, the model still fails to identify the most favourable areas (Supplementary Materials S2) correctly. This suggests that there are some relevant factors for the distribution of *Chalcides bedriagai* that have not been included in the modelling procedure. However, the calibration plot of step eight for *Sylvia conspicillata* shows that there is little room for improvement in the calibration of the model. We can consider that the model for *Sylvia conspicillata* produced at this step is a good model that reflects the entropy of the distribution of the species, even if the AUC value for this species (0.779) is lower than that of *Chalcides bedriagai* (0.789). Therefore, the performance of models based on AUC has to be always compared with the calibration [56] and entropy of the species to permit differentiation between good or bad models.

Shannon entropy and fuzzy entropy differ not only on the calculation but also on assumptions and mathematical constraints [16,22]. Shannon entropy has to be calculated on probabilities, but the sum of these probabilities in the system or study area must sum to 1 [17]. However, in species distribution models, the sum of probabilities of presence in each locality throughout a study area, estimated, for instance, with logistic regression, has to be equal to the number of presences of the species. This is why narrowly distributed species have low probability values sometimes, even in cells where the species are present, and common species have high values throughout the study area [24]. Therefore, assuming that the sum of probabilities has to be 1 resolves this inequality, but this is equivalent to assuming that the species is only present in one cell. Thus, the use of Shannon entropy in species distribution models can be justified mathematically, but it has no biogeographical justification. For this reason, Shannon entropy is commensurable between species only if models are performed with the same number of cells (as maximum entropy is equal to *log (number of cells)*).

An effective way to avoid using probability is to remove the effect of the prevalence from the model output. This is exactly what the favourability function does, and therefore favourability values are always commensurable between species [23]. Additionally, favourable areas obtained through the favourability function constitute a fuzzy system [23] and, therefore, favourability values can be used to calculate the fuzzy entropy (whose requirement is that it has to be applied to fuzzy systems). When fuzzy entropy is calculated, there is no restriction on the sum of favourabilities. Another advantage of the fuzzy entropy is that it always has values between zero and one, which produces results that are commensurable between species even if the entropy is calculated in different study areas (neither Shannon entropy nor the AIC are comparable for all species and systems). The fact that AUC values are more correlated with fuzzy entropy than with Shannon entropy is an additional indication that fuzzy entropy is more in line with the properties that biogeographical modellers try to measure with species distribution models.

Boltzmann [57,58,59,60] developed the statistical concept of entropy as the criterion for measuring the deviation of an observed frequency distribution from the homogeneous distribution [61]. The smaller the deviation between the two, the greater the entropy. The local favourability value indicates the degree to which the local probability value deviates from the expected homogeneous probability under the assumption of maximum entropy, which is given by the prevalence of the species in the territory. Therefore, the distribution of favourability throughout a territory represents the distribution of the deviation between the distribution of local probability and that of maximum entropy, fitting the criterion developed by Boltzmann.

The form of the stepwise reduction of the fuzzy entropy could also be helpful to distinguish the entropy due to a failing model from that due to a generalist species distribution. Our results showed that the stepwise modelling entails a reduction of fuzzy entropy; more specifically, a reduction of entropy tends to be produced in each step of the model with respect to the previous step. This could be expected because the concept of AIC is derived from entropy [19], but a reduction of entropy is also obtained when the stepwise approach is based on significance testing rather than on AIC (results not shown). In other words, the stepwise procedure reduces the fuzzy entropy in each of the steps, i.e., in step 0, we have maximum uncertainty as no variable is included (Figure 2 and Figure 3); in step 1, we have the entropy obtained with just one variable, and so on, and this reduction tends to proceed asymptotically (Figure 1). The fuzzy entropy of the models above this asymptote could be attributed to model deficiency, as the species is the same along the stepwise procedure and, thus, possesses the same distribution entropy in all the steps. However, the fact that from certain steps, the increase in complexity of the model entails practically no reduction of entropy indicates that the remaining entropy is mostly intrinsic to the species distribution rather than to the wrong model specification. This constitutes a new valuable finding, as this distinction cannot be made with classification or discrimination metrics, such as the AUC.

A step could yield a model with an entropy slightly higher than that of the previous step, although the AIC decreases (Table 2 and Table 3 and Supplementary Materials S1). Thus, even if the AIC is related to entropy, they are not exactly the same. In those cases, the fuzzy entropy could be designated as a new approach to select the final model, i.e., constructing the final model with the variables that consecutively produce a reduction of the fuzzy entropy in each of the steps. In our example species, this would mean that, according to the fuzzy entropy, the model for *Algyroides marchi* could finish on step 11, whereas the model of *Sylvia conspicillata* could finish on step 8. In both cases, favourable areas obtained in those steps are very similar to favourable areas obtained with all the AIC steps (Figure 2 and Figure 3), as the variables of the final steps are those defining just fine-scale distribution patterns [1], but, when producing models with higher entropy and lower parsimony, these last steps may be (slightly) counterproductive. Also, models tend to be better calibrated at the step with the lowest fuzzy entropy than at the final step (see, for instance, calibration plots for *Iberolacerta bonnali*, *Chersophilus duponti* or *Sylvia conspicillata* in Supplementary Materials S1). We are not suggesting that selection of variables has to be always performed by taking into account the entropy of the models, but this could be a possibility if the aim is to reduce the uncertainty with the minimum number of variables, obtaining highly efficient models.

## 5. Conclusions

We suggest using the favourability function and the fuzzy entropy to assess the uncertainty of the models. Fuzzy entropy does not have anything that makes it worse than Shannon entropy or AIC, but it has the advantage that it is comparable for all species and all systems, while neither Shannon nor AIC are. Furthermore, Shannon entropy is not appropriate when dealing with species distribution models. The stepwise selection of variables tends to produce increasingly complex and less parsimonious models that are simultaneously less uncertain [18]. Therefore, this approach implies a trade-off between parsimony and uncertainty in species distribution models. In stepwise modelling, each step yields a model with its own fuzzy entropy value and, thus, selecting the model with the lowest fuzzy entropy implies combining maximum reduction of uncertainty and maximum parsimony, which results in high efficiency. The form of the stepwise reduction of fuzzy entropy allows distinguishing the fraction of entropy attributable to the species distribution from that attributable to inefficient model specification, which is impossible to disentangle by analysing only the AUC of the model. Here we used some Spanish vertebrates as example species, but the methodology can be applied to any other living form whose distribution is known and to any other territory.

## Figures and Tables

**Table 1:** Species analysed in the present study. N is the number of presences in the study area. The number of steps of the stepwise approach and the step with the lowest fuzzy entropy (R) are indicated.

Group	Species	Species Code	N	Total Steps	Step Lowest R
Amphibians	*Chioglossa lusitanica*	Chi_lus	168	11	9
	*Euproctus asper*	Eup_asp	189	12	12
	*Triturus pygmaeus*	Tri_pyg	470	11	11
	*Alytes dickhilleni*	Aly_dic	135	12	12
	*Rana pyrenaica*	Ran_pyr	24	10	10
Reptiles	*Chalcides bedriagai*	Cha_bed	605	13	13
	*Algyroides marchi*	Alg_mar	30	12	11
	*Iberolacerta bonnali*	Ibe_bon	25	10	5
	*Lacerta schreiberi*	Lac_sch	576	15	13
Birds	*Aquila adalberti*	Aqu_ada	158	13	12
	*Caprimulgus ruficollis*	Cap_ruf	1725	13	13
	*Chersophilus duponti*	Che_dup	237	13	6
	*Sylvia conspicillata*	Syl_con	1299	10	8
Mammals	*Galemys pyrenaicus*	Gal_pyr	513	13	13
	*Lynx pardinus*	Lyn_par	29	8	7
	*Capra pyrenaica*	Cap_pyr	647	18	18
	*Microtus cabrerae*	Mic_cab	272	11	7
	*Lepus castroviejoi*	Lep_cas	69	10	6

**Table 2:** Steps of the model of *Algyroides marchi*. A “+” in the column Variable means the inclusion of the variable, a “−“ means that the variable was removed in that step. Cor_P is the Pearson correlation between the probabilities at each step and those of the final probability model. H is the Shannon entropy (obtained after dividing the probabilities by the number of presences). Cor_F is the Pearson correlation between the favourability values at each step and those of the final favourability model. R is the fuzzy entropy. AIC: Akaike Information Criterion. The row highlighted in blue indicates the step with the lowest fuzzy entropy. Variable codes as in Table S1.

Step	Variable	Cor_P	H	Cor_F	R	AIC
0	Intercept	-	8.58	-	1	372.38
1	+Calc	0.169	7.39	0.224	0.1897	302.57
2	+U500	0.621	5.61	0.406	0.0729	190.01
3	+AET	0.7396	5.19	0.446	0.0618	161.07
4	+Alt	0.837	4.85	0.468	0.0469	137.66
5	+SISSum	0.884	4.54	0.638	0.0465	116.82
6	+Grav	0.908	4.43	0.692	0.0391	110.61
7	+CTI	0.927	4.37	0.721	0.0378	107.92
8	+Sil	0.9395	4.28	0.839	0.0265	104.18
9	+U100	0.971	4.14	0.906	0.0119	96.53
10	+DTn20Aut	0.994	4.090	0.990	0.00871	94.47
11	−Calc	0.996	4.095	0.992	0.00848	92.91
12	−Alt	1	4.12	1	0.00863	92.08

**Table 3:** Steps of the model of *Sylvia conspicillata*. A “+” in the column Variable means the inclusion of the variable, a “−“ means that the variable was removed in that step. Cor_P is the Pearson correlation between the probabilities at each step and those of the final probability model. H is the Shannon entropy (obtained after dividing the probabilities by the number of presences). Cor_F is the Pearson correlation between the favourability values at each step and those of the final favourability model. R is the fuzzy entropy. AIC: Akaike Information Criterion. The row highlighted in blue indicates the step with the lowest fuzzy entropy. Variable codes as in Table S1.

Step	Variable	Cor_P	H	Cor_F	R	AIC
0	Intercept	-	8.58	-	1	5909.44
1	+DP10Aut	0.824	8.35	0.878	0.467	5160.62
2	+CTI	0.891	8.32	0.916	0.451	5063.95
3	+Sil	0.913	8.31	0.943	0.429	5021.69
4	+PSum	0.932	8.30	0.955	0.425	4994.18
5	+Alt	0.957	8.29	0.968	0.406	4955.96
6	+DHi	0.972	8.28	0.980	0.400	4929.42
7	+U500	0.983	8.276	0.989	0.3960	4911.05
8	+TRan	0.993	8.272	0.995	0.394	4894.74
9	+PET	0.997	8.271	0.998	0.395	4888.98
10	+Grav	1	8.270	1	0.3957	4885.93

## Data Availability

The data presented in this study are openly available in the UMA repository at https://dx.doi.org/10.24310/riuma.22488 (accessed on 2 August 2021).

## Supplementary Materials

The following are available online at https://www.mdpi.com/article/10.3390/e23081014/s1, Table S1: Environmental variables considered, Supplementary Materials S1: Complete results for all studied species, Supplementary Materials S2: Calibration plots of each step of the models of *Chalcides bedriagai* and *Sylvia conspicillata*.
