# Supplementary material for: A Stepwise Assessment of Parsimony and Fuzzy Entropy in Species Distribution Modelling
Source: Entropy (Basel). 2021 Aug 5;23(8):1014. doi: 10.3390/e23081014 (PMC8392680; doi:10.3390/e23081014)

A stepwise assessment of parsimony and fuzzy entropy in species distribution modelling

Alba Estrada & Raimundo Real

Supplementary Materials S1.

For each of the studied **species** we show the **entropy** of each of the step of the models. Variable codes as in Table S1; entropy codes as in Table 2. The row highlighted in blue indicates the step with the lowest fuzzy entropy. We also show **favourability models**, their **evaluation metrics** and the **calibration plot** of the step with the **lowest fuzzy entropy** as well as of the **last step** (final model). β: coefficients; SE: standard errors; Sig: significance: ***<0.001, **<0.01, *<0.05, ns>0.05; AUC: Area Under the Curve of the Receiver Operating Characteristic; CCR: Correct Classification Rate; HL: Hosmer and Lemeshow test. Numbers in the calibration plots indicate the sample size of each bin. The **maps** in each species are the **distribution** of the species in mainland Spain, **favourability** model of the step with **lowest fuzzy entropy**, and **final favourability** model. Colour ramp as in figure 1. Only one model is shown when the step with the lowest fuzzy entropy is the final step.

Chioglossa lusitanica

Entropy of each step:

Step	Variable	Cor_P	H	Cor_F	R	AIC
0	Intercept	-	8.58	-	1	1492.84
1	+ DP1Win	0.812	6.64	0.882	0.0965	773.32
2	+ Sil	0.887	6.47	0.894	0.0860	712.08
3	+ TRan	0.930	6.36	0.929	0.0761	662.97
4	+ DHi	0.944	6.32	0.940	0.0673	651.71
5	+ DTn20	0.947	6.27	0.962	0.0505	641.66
6	+ DTx25Spr	0.959	6.23	0.976	0.0481	626.74
7	+ SE	0.970	6.21	0.983	0.0443	619.32
8	+ DTn0Win	0.983	6.20	0.991	0.0477	614.23
9	+ CTI	0.997	6.167	0.997	0.0427	606.35
10	- TRan	0.999	6.174	0.997	0.0441	606.30
11	+ Clay	1	6.170	1	0.0439	606.18

Favourability model of step 9:

Variables	ß	SE	Sig
Intercept	13.005	3.49	**
DP1Win	0.064	0.024	**
Sil	3.733	0.454	***
TRan	-0.176	0.126	ns
DHi	-0.0203	0.00679	**
DTn20	-0.528	0.156	***
DTx25Spr	0.118	0.0455	**
SE	-0.0269	0.0105	*
DTn0Win	-0.0516	0.0142	***
CTI	-0.602	0.197	**

Evaluation metrics	
AUC	0.982
Kappa	0.431
Sensitivity	0.982
Specificity	0.925
CCR	0.927
HL	5.39 ns

Chioglossa lusitanica (cont.)

Final favourability model (step 11):

Variables	ß	SE	Sig	Evaluation metrics	
Intercept	12.07	3.48	*	AUC	0.982
DP1Win	0.0806	0.0231	***	Kappa	0.432
Sil	4.08	0.535	***	Sensitivity	0.964
DHi	-0.020	0.00675	**	Specificity	0.927
DTn20	-0.588	0.157	***	CCR	0.928
DTx25Spr	0.0899	0.0390	*	HL	10.12 ns
SE	-0.0274	0.0105	**		
DTn0Win	-0.0685	0.00895	***		
CTI	-0.689	0.191	***		
Clay	1.744	1.127	ns		

Calibration plots:

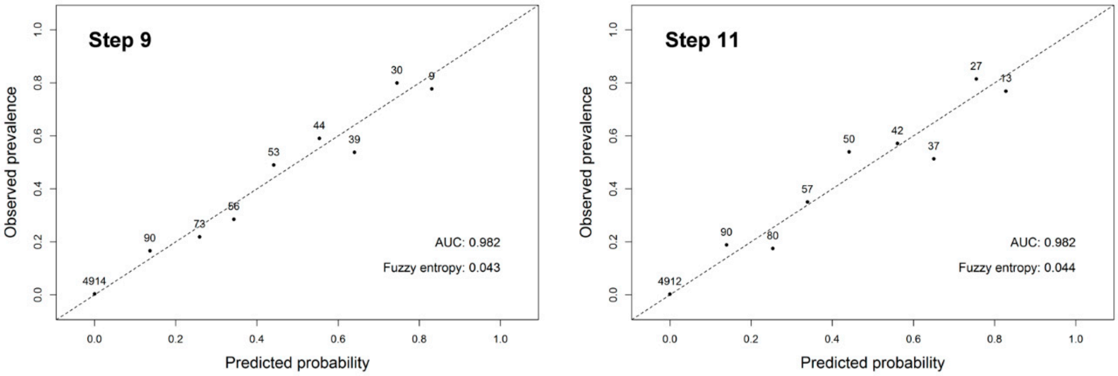

Distribution and favourability:

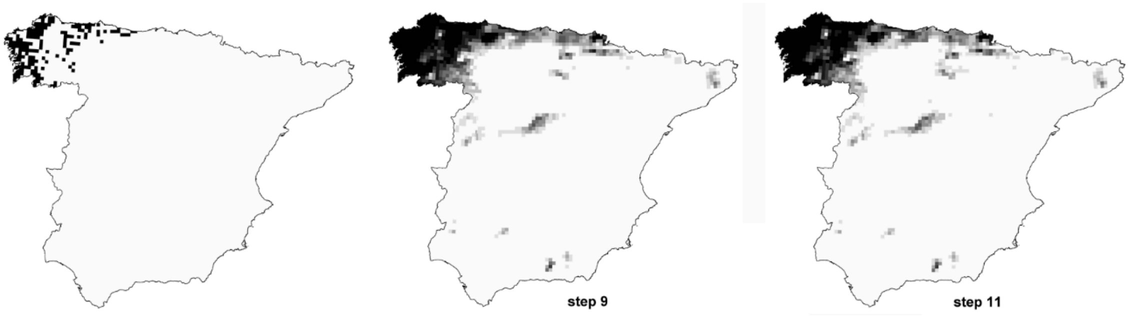

Euproctus asper

Entropy of each step:

Step	Variable	Cor_P	H	Cor_F	R	AIC
0	Intercept	-	8.58	-	1	1633.90
1	+ DP10Sum	0.880	6.49	0.799	0.114	709.36
2	+ TRan	0.943	6.34	0.878	0.122	622.89
3	+ HPd	0.964	6.27	0.904	0.106	595.06
4	+ AET	0.969	6.20	0.944	0.0893	571.64
5	+ DTn20	0.980	6.15	0.950	0.0770	555.61
6	+ DTn0Sum	0.984	6.12	0.960	0.0712	547.71
7	+ U500	0.985	6.10	0.974	0.0661	543.22
8	+ CTI	0.990	6.08	0.984	0.0619	536.99
9	+ SE	0.994	6.07	0.990	0.0608	532.26
10	+ DHi	0.996	6.065	0.993	0.0606	530.59
11	+ Sil	0.998	6.062	0.997	0.0611	529.15
12	+ Calc	1	6.05	1	0.0586	527.70

Final favourability model (step 12):

Variables	ß	SE	Sig
Intercept	-9.19	5.42	*
DP10Sum	1.04	0.088	***
TRan	0.351	0.0755	***
HPd	-0.0552	0.0202	**
AET	0.00950	0.00240	***
DTn20	-0.129	0.0347	***
DTn0Sum	-1.15	0.304	***
U500	-0.0132	0.00354	***
CTI	-0.607	0.294	*
SE	0.0340	0.0122	**
DHi	0.0145	0.00802	ns
Sil	1.38	0.541	*
Calc	1.03	0.567	ns

Evaluation metrics	
AUC	0.991
Kappa	0.534
Sensitivity	0.995
Specificity	0.942
CCR	0.944
HL	72.80 ***

Euproctus asper (cont.)

Calibration plot:

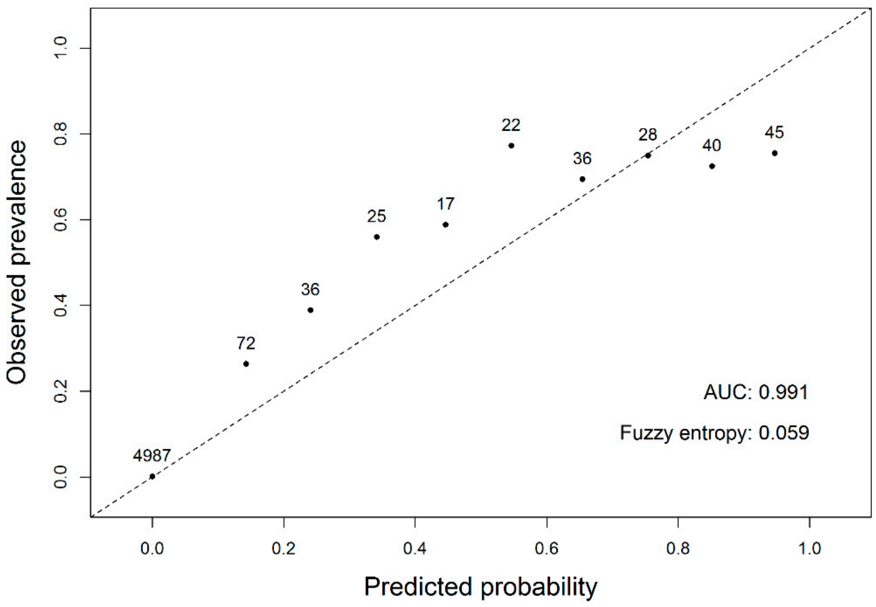

Distribution and favourability:

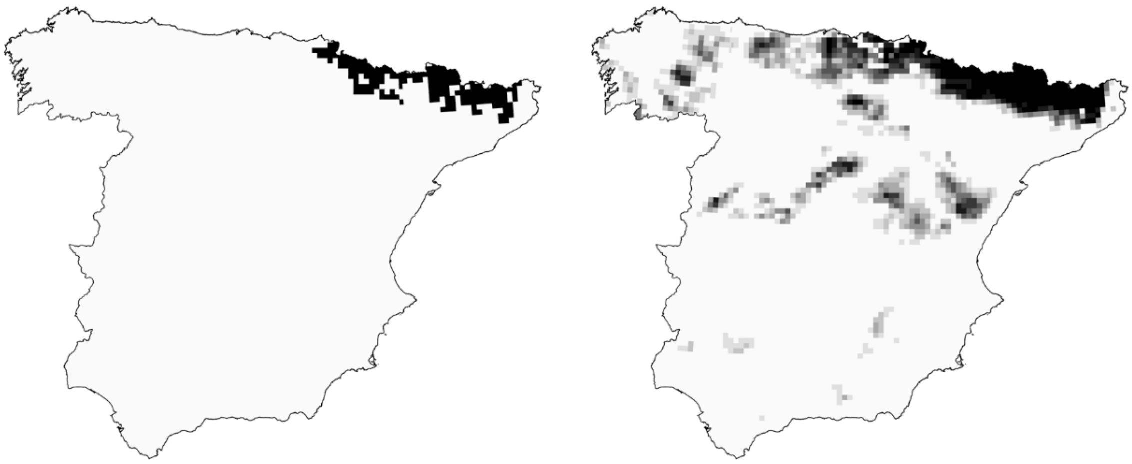

Triturus pygmaeus

Entropy of each step:

Step	Variable	Cor_P	H	Cor_F	R	AIC
0	Intercept	-	8.58	-	1	3177.88
1	+ SIDSum	0.753	7.79	0.892	0.216	2362.04
2	+ DP30Win	0.901	7.61	0.960	0.174	2145.98
3	+ Sil	0.943	7.56	0.973	0.166	2098.12
4	+ Calc	0.956	7.55	0.978	0.165	2081.20
5	+ SE	0.966	7.54	0.983	0.167	2069.31
6	+ DTn20	0.971	7.53	0.987	0.162	2061.44
7	+ PSpr	0.978	7.52	0.990	0.159	2054.13
8	+ TRan	0.993	7.50	0.995	0.1523	2038.54
9	+ DP30Sum	0.997	7.497	0.997	0.1516	2033.76
10	+ DTn0Spr	0.9998	7.4925	0.9999	0.1502	2030.00
11	- DP30Win	1	7.4923	1	0.1498	2028.31

Final favourability model (step 11):

Variables	ß	SE	Sig
Intercept	-29.19	2.55	***
SIDSum	4.99	0.450	***
Sil	1.25	0.180	***
Calc	1.16	0.276	***
SE	0.0152	0.00475	**
DTn20	0.0105	0.00488	*
PSpr	0.0169	0.00163	***
TRan	-0.163	0.0374	***
DP30Sum	-1.65	0.644	*
DTn0Spr	-0.0360	0.0142	*

Evaluation metrics	
AUC	0.912
Kappa	0.355
Sensitivity	0.917
Specificity	0.783
CCR	0.795
HL	21.69 **

Triturus pygmaeus (cont.)

Calibration plot:

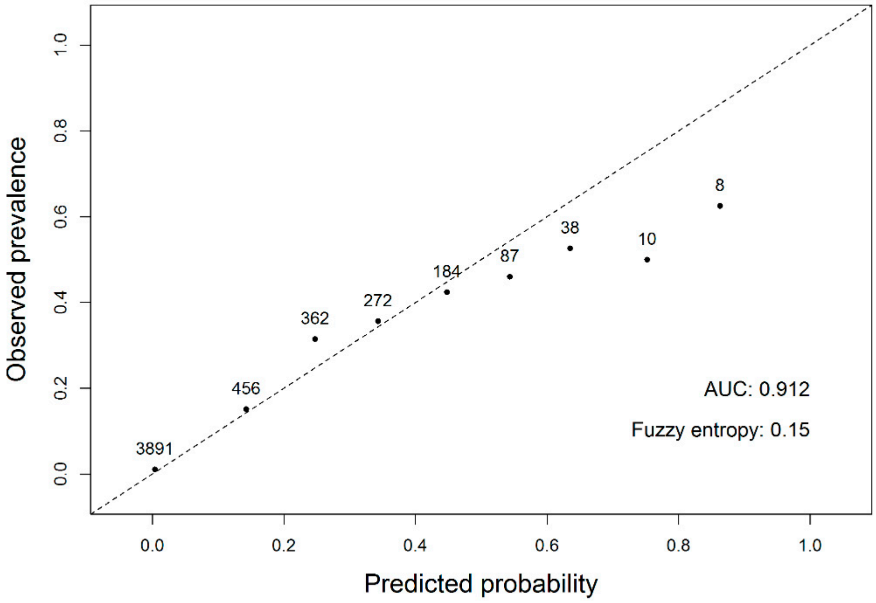

Distribution and favourability:

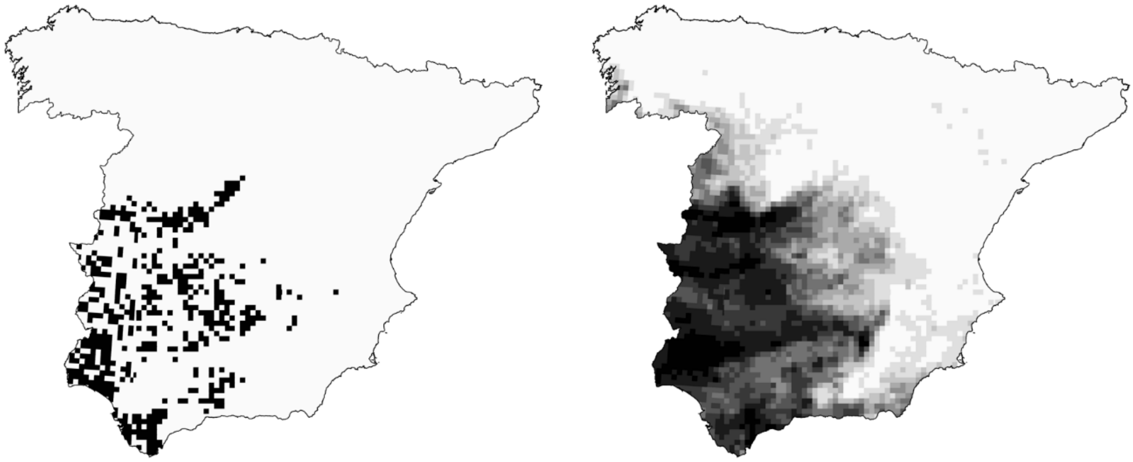

Alytes dickhilleni

Entropy of each step:

Step	Variable	Cor_P	H	Cor_F	R	AIC
0	Intercept	-	8.58	-	1	1259.89
1	+ Slop	0.322	7.88	0.353	0.428	1060.62
2	+ SISWin	0.578	7.10	0.668	0.219	828.64
3	+ Alt	0.731	6.66	0.818	0.157	684.53
4	+ Sil	0.864	6.33	0.876	0.0985	590.49
5	+ DHi	0.917	6.15	0.929	0.0627	539.46
6	+ TRan	0.935	6.09	0.947	0.0589	520.94
7	+ TnWin	0.973	6.01	0.963	0.0572	491.15
8	+ U500	0.977	5.99	0.976	0.0580	487.91
9	+ AET	0.987	5.97	0.990	0.0579	480.31
10	+ U100	0.992	5.95	0.994	0.0540	477.29
11	+ Clay	0.997	5.94	0.997	0.0531	475.67
12	+ DP30Sum	1	5.93	1	0.0507	474.28

Final favourability model (step 12):

Variables	ß	SE	Sig		Evaluation metrics	
Intercept	-56.8	6.34	***		AUC	0.987
Slop	0.340	0.0556	***		Kappa	0.434
SISWin	12.31	1.63	***		Sensitivity	0.985
Alt	0.00663	0.000949	***		Specificity	0.939
Sil	-3.09	0.567	***		CCR	0.940
DHi	0.0477	0.0121	***		HL	18.15 *
TRan	0.600	0.131	***			
TnWin	0.536	0.149	***			
U500	0.0142	0.00354	***			
AET	0.00441	0.00132	***			
U100	-0.0151	0.00728	*			
Clay	0.986	0.493	*			
DP30Sum	1.09	0.588	ns			

Alytes dickhilleni (cont.)

Calibration plot:

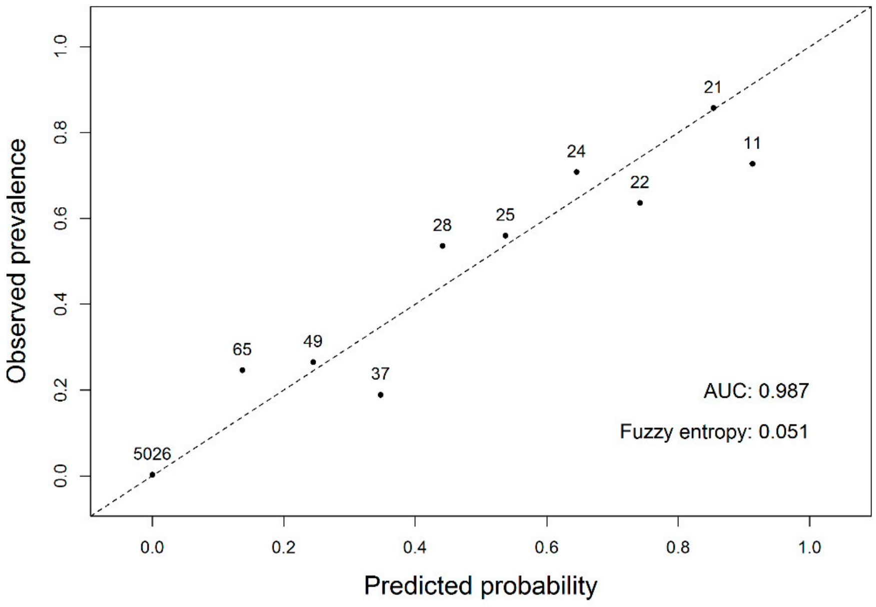

Distribution and favourability:

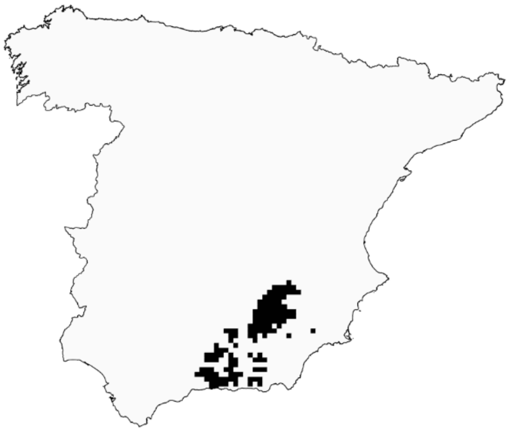

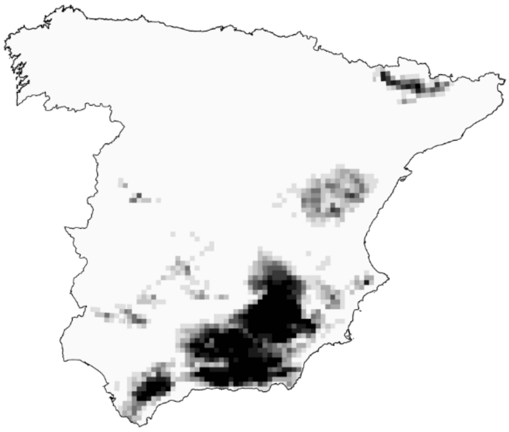

Rana pyrenaica

Entropy of each step:

Step	Variable	Cor_P	H	Cor_F	R	AIC
0	Intercept	-	8.58	-	1	309.04
1	+ DP10Sum	0.537	5.69	0.513	0.0996	167.66
2	+ HPd	0.627	5.21	0.620	0.0456	144.84
3	+ Calc	0.828	4.86	0.630	0.0357	125.24
4	+ DP10Spr	0.921	4.61	0.666	0.0280	112.21
5	+ U100	0.945	4.47	0.728	0.0270	103.68
6	+ TxAut	0.960	4.37	0.773	0.0220	101.06
7	+ AET	0.976	4.29	0.803	0.0248	97.44
8	+ Grav	0.985	4.20	0.935	0.0198	94.12
9	+ DHi	0.992	4.15	0.970	0.0170	93.66
10	+ Clay	1	4.11	1	0.0159	93.19

Final favourability model (step 10):

Variables	ß	SE	Sig		Evaluation metrics	
Intercept	-24.50	7.65	***		AUC	0.997
DP10Sum	0.695	0.297	*		Kappa	0.243
HPd	-0.989	0.463	*		Sensitivity	0.958
Calc	3.25	0.907	***		Specificity	0.974
DP10Spr	0.755	0.185	***		CCR	0.974
U100	0.0796	0.0232	***		HL	6.82 ns
TxAut	0.855	0.268	**			
AET	-0.0143	0.00625	*			
Grav	-0.000243	0.0000121	ns			
DHi	0.0413	0.0264	ns			
Clay	-5.93	4.92	ns			

Rana pyrenaica (cont.)

Calibration plot:

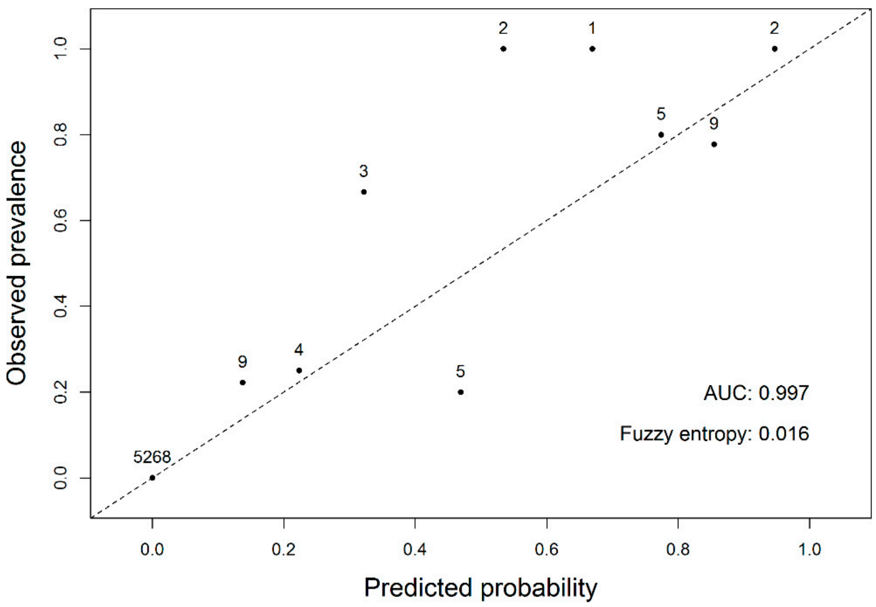

Distribution and favourability:

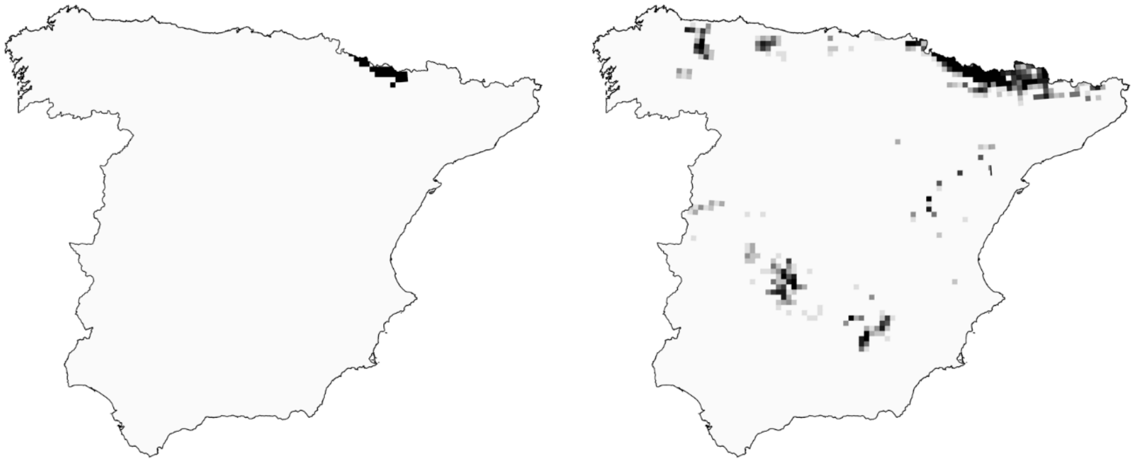

Chalcides bedriagai

Entropy of each step:

Step	Variable	Cor_P	H	Cor_F	R	AIC
0	Intercept	-	8.58	-	1	3768.07
1	+ SISWin	0.688	8.36	0.691	0.513	3470.59
2	+ TRan	0.831	8.29	0.807	0.497	3357.67
3	+ Clay	0.897	8.25	0.856	0.473	3304.01
4	+ Alt	0.919	8.23	0.896	0.463	3274.26
5	+ U500	0.952	8.20	0.932	0.440	3239.23
6	+ Gyps	0.955	8.192	0.953	0.432	3229.89
7	+ PSpr	0.967	8.185	0.964	0.429	3219.77
8	+ TxJan	0.977	8.175	0.980	0.422	3208.53
9	+ U100	0.985	8.168	0.988	0.414	3201.58
10	+ Grav	0.989	8.165	0.991	0.4130	3199.49
11	+ DTn20	0.994	8.164	0.992	0.415	3197.97
12	+ DTn0Sum	0.997	8.161	0.997	0.4135	3195.70
13	+ DAlt	1	8.159	1	0.411	3194.63

Final favourability model (step 13):

Variables	ß	SE	Sig
Intercept	-0.417	0.962	*
SISWin	1.04	0.441	*
TRan	-0.271	0.0371	***
Clay	-1.57	0.206	***
Alt	0.00241	0.000387	***
U500	-0.00724	0.000910	***
Gyps	-2.59	1.22	*
PSpr	-0.00489	0.00131	***
TxJan	0.244	0.0713	***
U100	0.00487	0.00205	*
Grav	-0.429	0.227	ns
DTn20	0.0103	0.00402	*
DTn0Sum	-0.645	0.342	ns
DAlt	-0.000364	0.000209	ns

Evaluation metrics	
AUC	0.789
Kappa	0.263
Sensitivity	0.736
Specificity	0.734
CCR	0.734
HL	21.71 **

Chalcides bedriagai (cont.)

Calibration plot:

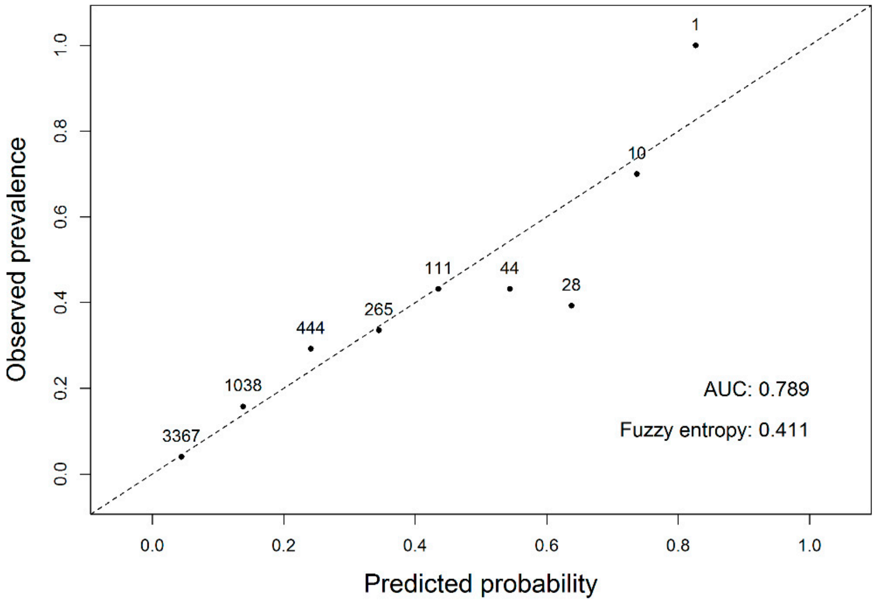

Distribution and favourability:

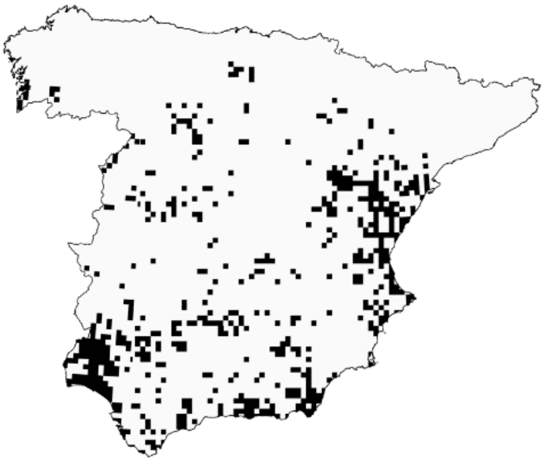

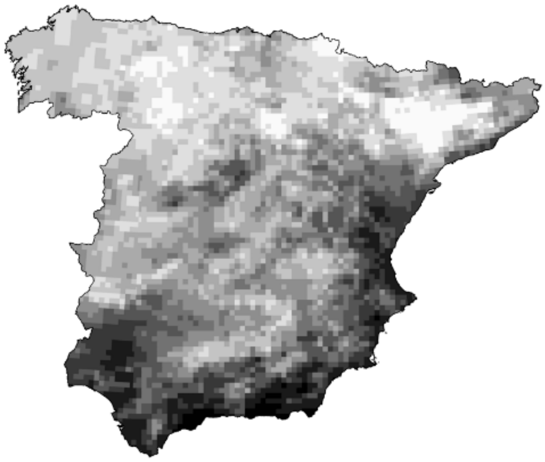

Algyroides marchi

Entropy of each step:

Step	Variable	Cor_P	H	Cor_F	R	AIC
0	Intercept	-	8.58	-	1	372.38
1	+ Calc	0.169	7.39	0.224	0.1897	302.57
2	+ U500	0.621	5.61	0.406	0.0729	190.01
3	+ AET	0.740	5.19	0.446	0.0618	161.07
4	+ Alt	0.837	4.85	0.468	0.0469	137.66
5	+ SISSum	0.884	4.54	0.638	0.0465	116.82
6	+ Grav	0.908	4.43	0.692	0.0391	110.61
7	+ CTI	0.927	4.37	0.721	0.0378	107.92
8	+ Sil	0.940	4.28	0.839	0.0265	104.18
9	+ U100	0.971	4.14	0.906	0.0119	96.53
10	+ DTn20Aut	0.994	4.090	0.990	0.00871	94.47
11	- Calc	0.996	4.095	0.992	0.00848	92.91
12	- Alt	1	4.12	1	0.00863	92.08

Favourability model of step 11:

Variables	ß	SE	Sig
Intercept	-20.54	18.57	ns
U500	0.0665	0.0181	***
AET	0.0103	0.00394	**
Alt	0.00156	0.00145	ns
SISSum	7.16	2.09	***
Grav	-38.74	28.06	ns
CTI	-2.69	0.878	**
Sil	-9.60	3.10	**
U100	-0.0541	0.0210	*
DTn20Aut	-0.689	0.402	ns

Evaluation metrics	
AUC	0.999
Kappa	0.433
Sensitivity	1
Specificity	0.985
CCR	0.985
HL	14.56 ns

Final favourability model (step 12):

Variables	ß	SE	Sig
Intercept	-18.32	17.01	ns
U500	0.0631	0.0168	***
AET	0.0101	0.00382	**
SISSum	7.70	1.90	***
Grav	-37.16	26.75	ns
CTI	-2.94	0.864	***
Sil	-10.55	3.29	**
U100	-0.0473	0.0195	*
DTn20Aut	-0.938	0.354	**

Evaluation metrics	
AUC	0.999
Kappa	0.433
Sensitivity	1
Specificity	0.985
CCR	0.985
HL	9.16 ns

Algyroides marchi (cont.)

Calibration plots:

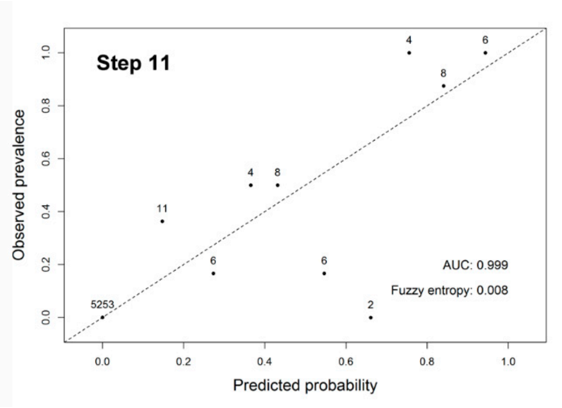

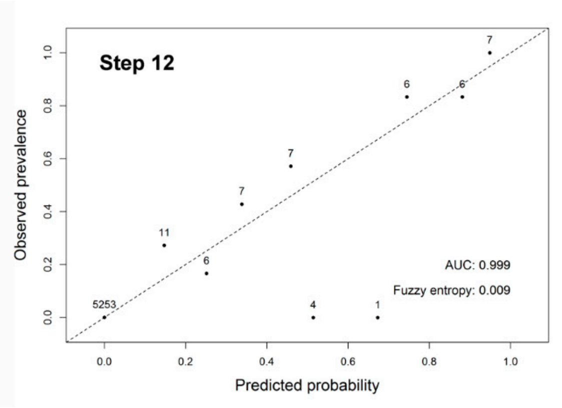

Distribution and favourability:

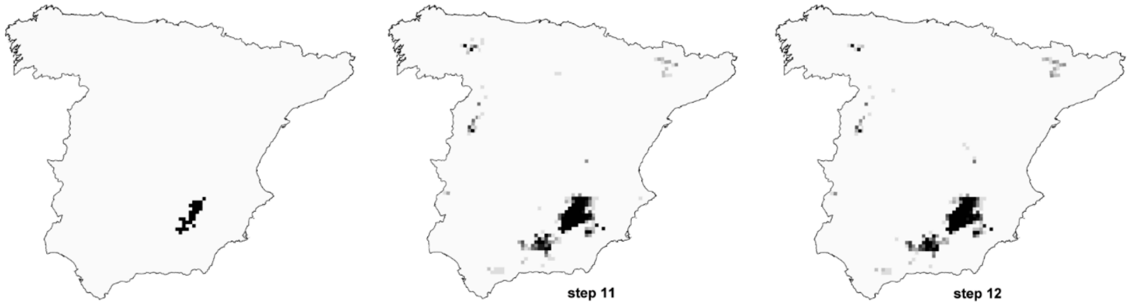

Iberolacerta bonnali

Entropy of each step:

Step	Variable	Cor_P	H	Cor_F	R	AIC
0	Intercept	-	8.58	-	1	319.79
1	+ TxAut	0.821	4.49	0.643	0.0340	98.48
2	+ DP10Sum	0.894	4.18	0.787	0.0198	83.36
3	+ SE	0.935	4.06	0.829	0.0150	77.71
4	+ U100	0.940	3.93	0.895	0.00632	71.64
5	+ Alt	0.955	3.85	0.910	0.00445	68.73
6	+ DTn0Sum	0.970	3.82	0.961	0.00637	66.22
7	- TxAut	0.972	3.83	0.962	0.00664	64.64
8	+ DHi	0.983	3.79	0.968	0.00586	64.48
9	+ Sil	0.994	3.77	0.985	0.00456	64.29
10	- U100	1	3.80	1	0.00565	64.00

Favourability model of step 5:

Variables	ß	SE	Sig	Evaluation metrics	
Intercept	-16.38	11.25	ns	AUC	0.999
TxAut	0.107	0.391	ns	Kappa	0.438
DP10Sum	1.83	0.532	***	Sensitivity	1
SE	-0.115	0.0369	**	Specificity	0.988
U100	0.0661	0.0260	*	CCR	0.988
Alt	0.00313	0.00173	ns	HL	4.23 ns

Final favourability model (step 10):

Variables	ß	SE	Sig	Evaluation metrics	
Intercept	-8.88	4.24	***	AUC	0.999
DP10Sum	1.10	0.329	***	Kappa	0.468
SE	-0.135	0.0408	***	Sensitivity	1
Alt	0.00352	0.00127	**	Specificity	0.989
DTn0Sum	2.93	0.914	**	CCR	0.989
DHi	0.0728	0.0366	*	HL	12.38 ns
Sil	2.22	1.38	ns		

Iberolacerta bonnali (cont.)

Calibration plots:

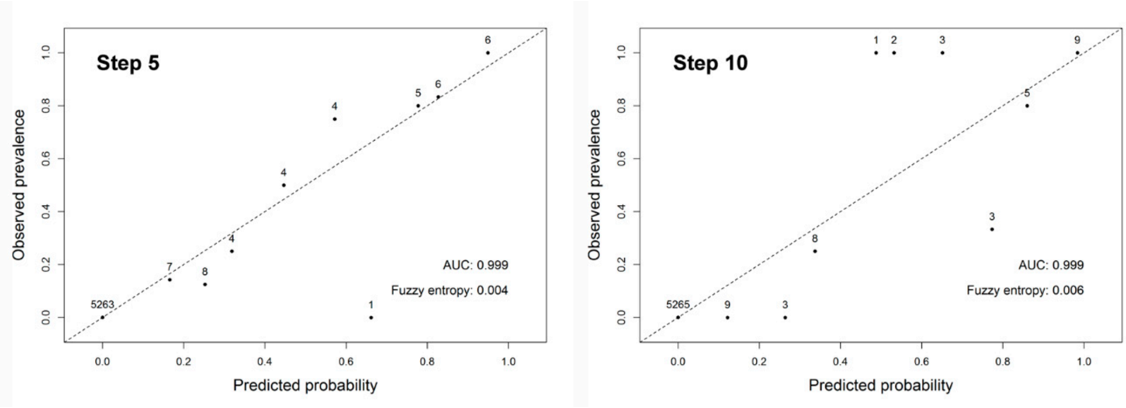

Distribution and favourability:

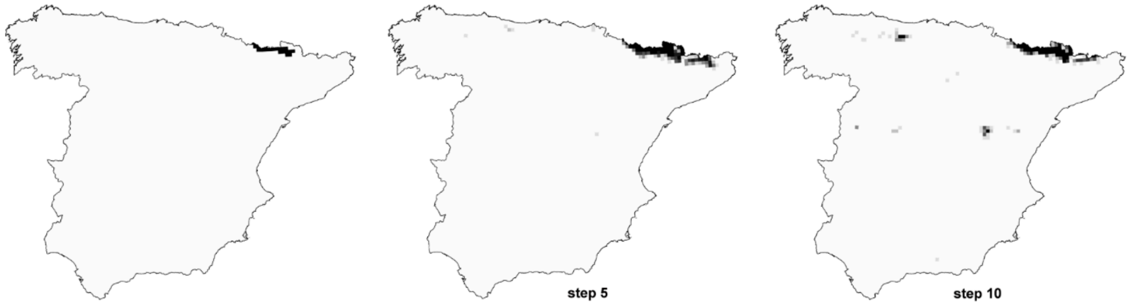

Lacerta schreiberi

Entropy of each step:

Step	Variable	Cor_P	H	Cor_F	R	AIC
0	Intercept	-	8.58	-	1	3647.54
1	+ DP1Win	0.820	7.68	0.840	0.234	2341.46
2	+ Sil	0.881	7.56	0.837	0.217	2175.65
3	+ PET	0.908	7.46	0.906	0.179	2049.41
4	+ AET	0.936	7.32	0.952	0.118	1908.60
5	+ DP30Sum	0.971	7.27	0.975	0.113	1821.27
6	+ DHi	0.978	7.26	0.983	0.111	1797.80
7	+ DP30Spr	0.985	7.25	0.987	0.109	1782.21
8	+ Alt	0.990	7.24	0.989	0.107	1772.64
9	+ Gyps	0.990	7.23	0.994	0.105	1767.40
10	+ DAlt	0.993	7.227	0.995	0.1037	1762.99
11	+ Calc	0.995	7.222	0.996	0.1036	1758.54
12	+ SE	0.997	7.218	0.997	0.1022	1754.82
13	+ DTn20	0.998	7.215	0.999	0.10178	1752.70
14	+ Clay	0.999	7.213	0.999	0.10184	1751.44
15	+ U100	1	7.212	1	0.10187	1751.20

Favourability model of step 13:

Variables	ß	SE	Sig
Intercept	6.34	1.49	**
DP1Win	0.0528	0.0121	***
Sil	1.23	0.238	***
PET	-0.0208	0.002	***
AET	0.0140	0.00159	***
DP30Sum	-1.73	0.197	***
DHi	-0.0137	0.00308	***
DP30Spr	0.399	0.0975	***
Alt	-0.000974	0.000253	***
Gyps	-62990.00	449900.00	ns
DAlt	0.000748	0.000242	**
Calc	-0.895	0.345	**
SE	-0.0113	0.00528	*
DTn20	-0.0224	0.0113	*

Evaluation metrics	
AUC	0.954
Kappa	0.547
Sensitivity	0.927
Specificity	0.866
CCR	0.873
HL	10.31 ns

Lacerta schreiberi (cont.)

Final favourability model (step 15):

Variables	ß	SE	Sig
Intercept	7.07	1.58	**
DP1Win	0.0503	0.0122	***
Sil	0.650	0.389	ns
PET	-0.0211	0.00203	***
AET	0.0140	0.00161	***
DP30Sum	-1.73	0.196	***
DHi	-0.0156	0.00332	***
DP30Spr	0.423	0.0987	***
Alt	-0.00103	0.000255	***
Gyps	-63930.00	451400.00	ns
DAlt	0.000801	0.000243	***
Calc	-1.40	0.450	**
SE	-0.0119	0.00529	*
DTn20	-0.0246	0.0118	*
Clay	-0.919	0.524	ns
U100	0.00375	0.00250	ns

Evaluation metrics	
AUC	0.954
Kappa	0.549
Sensitivity	0.927
Specificity	0.867
CCR	0.874
HL	11.89 ns

Calibration plots:

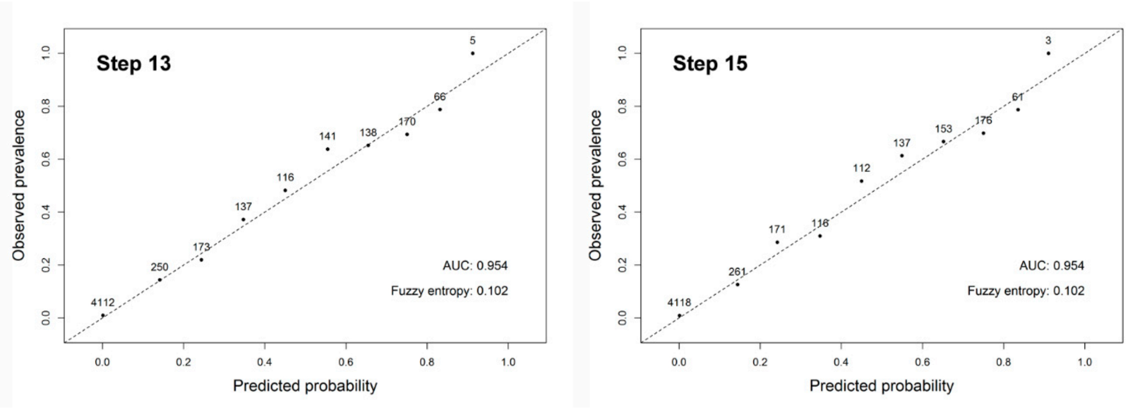

Distribution and favourability:

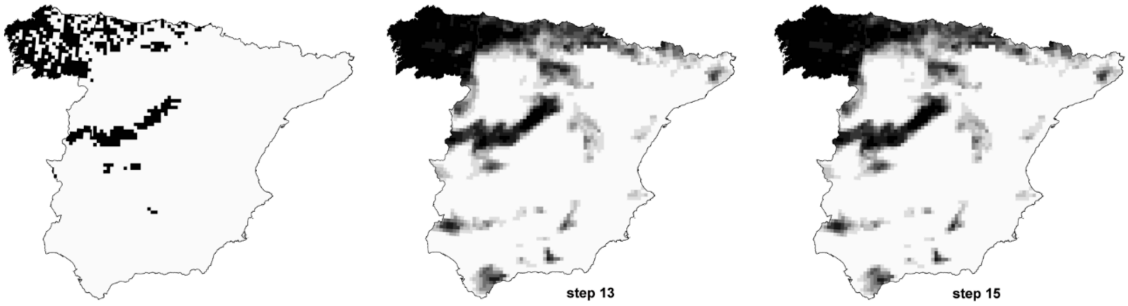

Aquila adalberti

Entropy of each step:

Step	Variable	Cor_P	H	Cor_F	R	AIC
0	Intercept	-	8.58	-	1	1423.79
1	+ SIDSum	0.485	7.96	0.725	0.291	1223.85
2	+ Sil	0.707	7.65	0.847	0.233	1123.37
3	+ TRan	0.812	7.53	0.896	0.213	1081.45
4	+ AET	0.880	7.47	0.901	0.203	1062.65
5	+ Calc	0.892	7.12	0.938	0.190	1047.84
6	+ DP30Sum	0.918	7.38	0.960	0.183	1036.32
7	+ SIDWin	0.945	7.35	0.965	0.182	1029.56
8	+ DTn20	0.967	7.33	0.975	0.171	1022.36
9	+ DP30Spr	0.985	7.31	0.980	0.174	1017.41
10	+ Grav	0.994	7.2897	0.991	0.1723	1012.33
11	- SIDSum	0.994	7.2895	0.991	0.1716	1010.38
12	+ Gyps	0.994	7.281	0.998	0.168	1009.64
13	+ DHi	1	7.277	1	0.1721	1009.37

Favourability model of step 12:

Variables	ß	SE	Sig
Intercept	-19.46	2.04	***
Sil	2.03	0.416	***
TRan	0.621	0.0765	***
AET	0.00665	0.000934	***
Calc	-2.33	0.757	**
DP30Sum	-1.65	0.816	*
SIDWin	2.76	0.618	***
DTn20	-0.0298	0.00994	**
DP30Spr	-0.671	0.249	**
Grav	1.74	0.652	**
Gyps	-40680.00	485400.00	ns

Evaluation metrics	
AUC	0.910
Kappa	0.150
Sensitivity	0.842
Specificity	0.791
CCR	0.792
HL	3.86 ns

Final favourability model (step 13):

Variables	ß	SE	Sig
Intercept	-18.90	2.03	***
Sil	2.13	0.422	***
TRan	0.613	0.0757	***
AET	0.00655	0.000934	***
Calc	-2.26	0.759	**
DP30Sum	-1.64	0.817	*
SIDWin	2.63	0.616	***
DTn20	-0.0293	0.00997	**
DP30Spr	-0.677	0.250	**
Grav	1.62	0.660	*
Gyps	-40490.00	486800.00	ns
DHi	-0.00591	0.00397	ns

Evaluation metrics	
AUC	0.910
Kappa	0.151
Sensitivity	0.861
Specificity	0.788
CCR	0.790
HL	2.51 ns

Aquila adalberti (cont.)

Calibration plots:

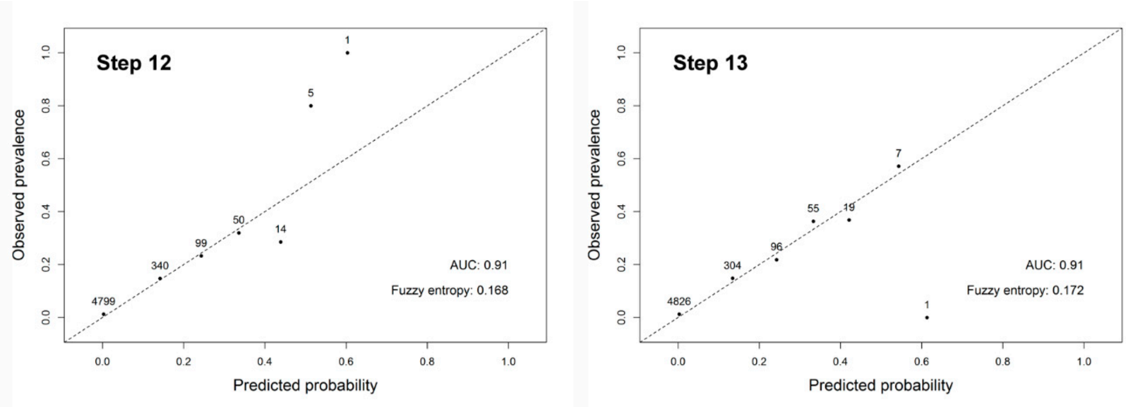

Distribution and favourability:

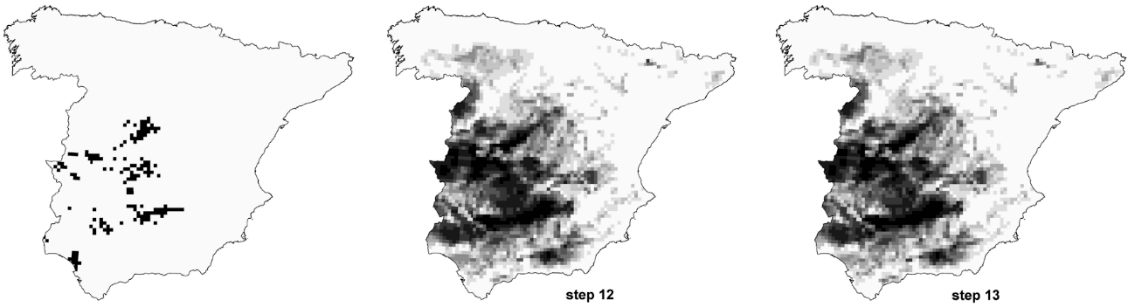

Caprimulgus ruficollis

Entropy of each step:

Step	Variable	Cor_P	H	Cor_F	R	AIC
0	Intercept	-	8.58	-	1	6696.10
1	+ DP01Sum	0.889	8.27	0.912	0.338	5172.94
2	+ U500	0.924	8.25	0.946	0.332	5049.78
3	+ TRan	0.956	8.22	0.963	0.307	4944.54
4	+ DTn0Spr	0.975	8.21	0.984	0.290	4863.48
5	+ U100	0.981	8.20	0.987	0.288	4846.61
6	+ DTx25Spr	0.986	8.195	0.990	0.285	4828.23
7	+ DHi	0.989	8.193	0.992	0.2818	4820.44
8	+ Gyps	0.991	8.192	0.994	0.2817	4815.57
9	+ SE	0.993	8.190	0.996	0.2803	4809.22
10	+ Calc	0.995	8.1899	0.997	0.2804	4804.16
11	+ Clay	0.997	8.1891	0.998	0.2801	4801.97
12	+ PSpr	0.998	8.187	0.999	0.279	4799.02
13	+ CTI	1	8.185	1	0.278	4794.28

Final favourability model (step 13):

Variables	ß	SE	Sig
Intercept	0.506	0.928	ns
DP01Sum	-0.203	0.0140	***
U500	-0.00627	0.000735	***
TRan	0.332	0.0258	***
DTn0Spr	-0.0603	0.00659	***
U100	-0.00530	0.00147	***
DTx25Spr	-0.0357	0.00763	***
DHi	-0.00544	0.00191	**
Gyps	-1.35	0.362	***
SE	0.00618	0.00268	*
Calc	-0.480	0.131	***
Clay	-0.351	0.144	*
PSpr	-0.00330	0.00110	**
CTI	-0.107	0.0412	**

Evaluation metrics	
AUC	0.834
Kappa	0.475
Sensitivity	0.857
Specificity	0.682
CCR	0.739
HL	21.11 **

Caprimulgus ruficollis (cont.)

Calibration plot:

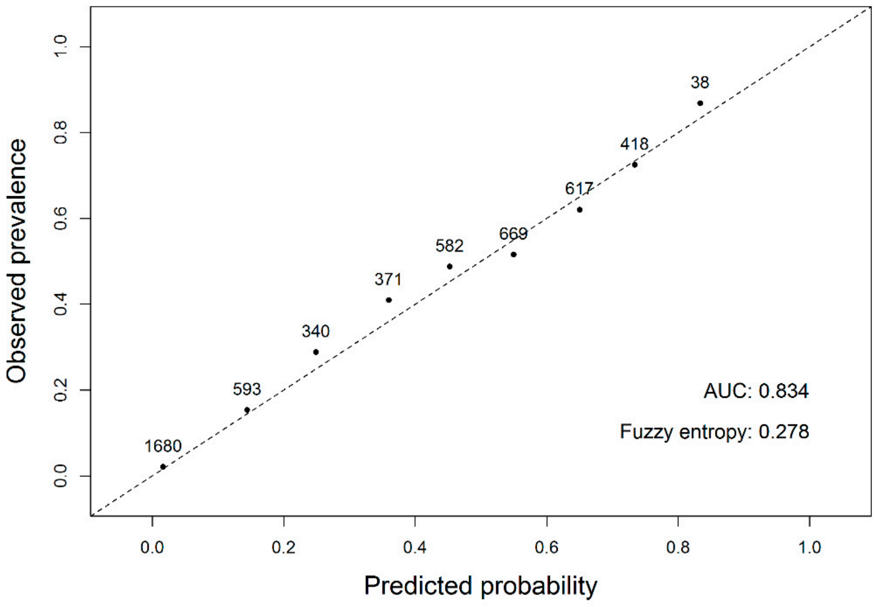

Distribution and favourability:

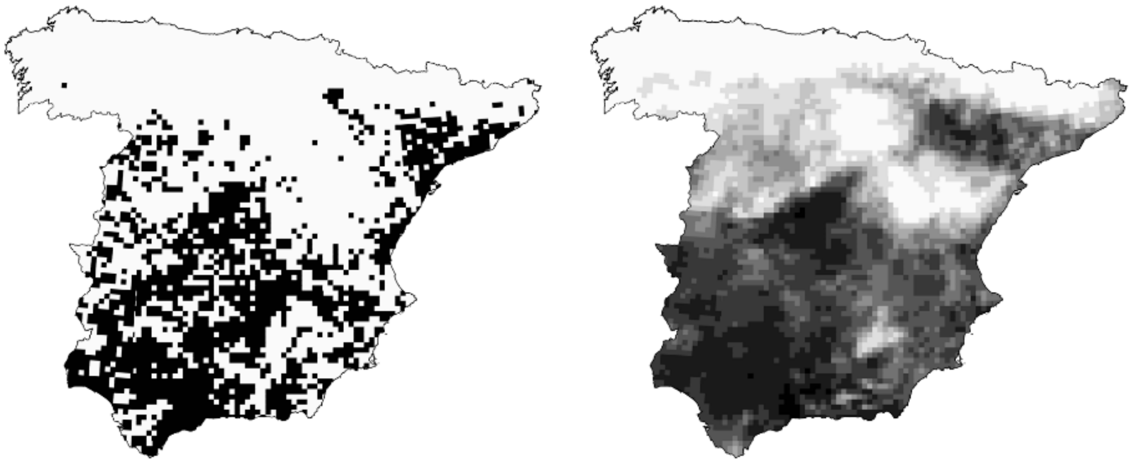

Chersophilus duponti

Entropy of each step:

Step	Variable	Cor_P	H	Cor_F	R	AIC
0	Intercept	-	8.58	-	1	1938.88
1	+ PWin	0.542	7.93	0.759	0.291	1618.35
2	+ PET	0.866	7.60	0.879	0.213	1442.39
3	+ U100	0.912	7.54	0.904	0.223	1407.87
4	+ U500	0.937	7.49	0.925	0.217	1384.53
5	+ Slop	0.950	7.46	0.945	0.205	1372.64
6	+ HPd	0.961	7.43	0.973	0.193	1358.84
7	+ WE	0.969	7.42	0.977	0.195	1353.92
8	+ DHi	0.979	7.416	0.981	0.202	1349.20
9	+ Alt	0.985	7.406	0.985	0.1987	1345.87
10	+ DTn20	0.989	7.397	0.990	0.1991	1342.85
11	+ TJan	0.994	7.386	0.994	0.1941	1339.61
12	+ AET	0.998	7.380	0.998	0.1942	1337.73
13	+ TRan	1	7.377	1	0.197	1337.45

Favourability model of step 6:

Variables	ß	SE	Sig	Evaluation metrics	
Intercept	11.46	1.05	***	AUC	0.894
PWin	-0.0237	0.00220	***	Kappa	0.178
PET	-0.00993	0.00119	***	Sensitivity	0.831
U100	0.0176	0.00283	***	Specificity	0.763
U500	-0.00956	0.00194	***	CCR	0.766
Slop	-0.199	0.0527	***	HL	14.22 *
HPd	-0.0147	0.00635	*		

Final favourability model (step 13):

Variables	ß	SE	Sig	Evaluation metrics	
Intercept	13.48	2.14	***	AUC	0.900
PWin	-0.0194	0.00285	***	Kappa	0.192
PET	-0.00507	0.00299	ns	Sensitivity	0.840
U100	0.0128	0.00343	***	Specificity	0.777
U500	-0.0124	0.00207	***	CCR	0.779
Slop	-0.315	0.0643	***	HL	19.49 **
HPd	-0.0118	0.00588	*		
WE	-0.0184	0.00651	**		
DHi	0.00792	0.00388	*		
Alt	0.000817	0.000515	ns		
DTn20	0.0319	0.0116	**		
TJan	-0.293	0.111	**		
AET	-0.00245	0.00121	*		
TRan	-0.134	0.0885	ns		

Chersophilus duponti (cont.)

Calibration plots:

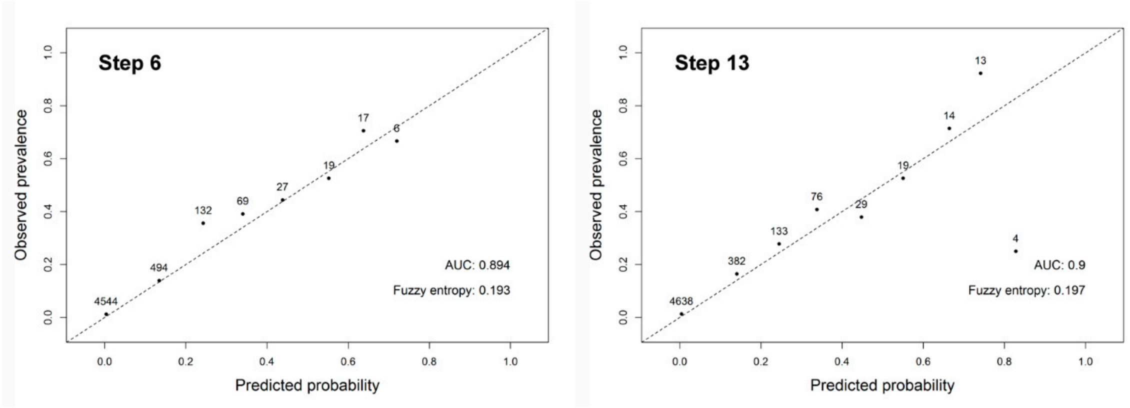

Distribution and favourability:

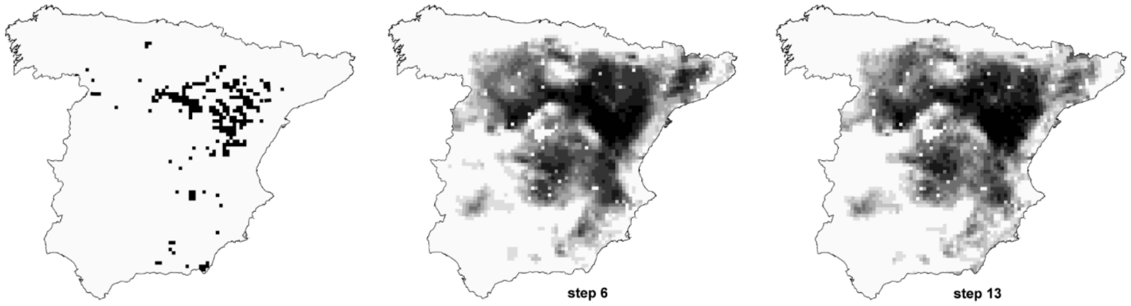

Sylvia conspicillata

Entropy of each step:

Step	Variable	Cor_P	H	Cor_F	R	AIC
0	Intercept	-	8.58	-	1	5909.44
1	+ DP10Aut	0.824	8.35	0.878	0.467	5160.62
2	+ CTI	0.891	8.32	0.916	0.451	5063.95
3	+ Sil	0.913	8.31	0.943	0.429	5021.69
4	+ PSum	0.932	8.30	0.955	0.425	4994.18
5	+ Alt	0.957	8.29	0.968	0.406	4955.96
6	+ DHi	0.972	8.28	0.980	0.400	4929.42
7	+ U500	0.983	8.276	0.989	0.3960	4911.05
8	+ TRan	0.993	8.272	0.995	0.394	4894.74
9	+ PET	0.997	8.271	0.998	0.395	4888.98
10	+ Grav	1	8.270	1	0.3957	4885.93

Favourability model of step 8:

Variables	ß	SE	Sig	Evaluation metrics	
Intercept	10.36	0.873	***	AUC	0.779
DP10Aut	-0.494	0.0293	***	Kappa	0.334
CTI	-0.311	0.0461	***	Sensitivity	0.791
Sil	-0.652	0.123	***	Specificity	0.643
PSum	-0.00877	0.00114	***	CCR	0.679
Alt	0.00122	0.000133	***	HL	6.90 ns
DHi	-0.00708	0.00184	***		
U500	-0.00343	0.000687	***		
TRan	-0.0931	0.0218	***		

Final favourability model (step 10):

Variables	ß	SE	Sig	Evaluation metrics	
Intercept	8.20	1.40	***	AUC	0.781
DP10Aut	-0.491	0.0295	***	Kappa	0.340
CTI	-0.306	0.0559	***	Sensitivity	0.796
Sil	-0.603	0.126	***	Specificity	0.646
PSum	-0.00658	0.00142	***	CCR	0.683
Alt	0.00155	0.000183	***	HL	24.01 **
DHi	-0.00633	0.00187	***		
U500	-0.00354	0.000691	***		
TRan	-0.0908	0.0229	***		
PET	0.00194	0.000746	**		
Grav	0.453	0.201	*		

Sylvia conspicillata (cont.)

Calibration plots:

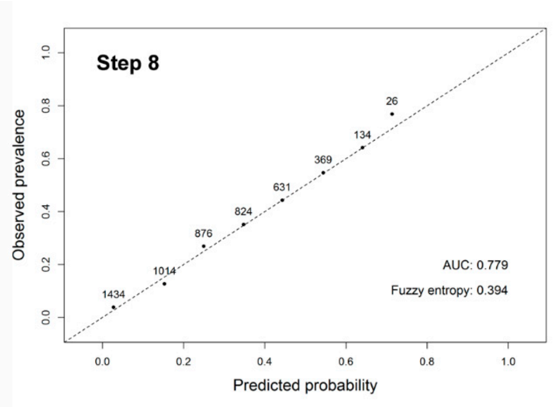

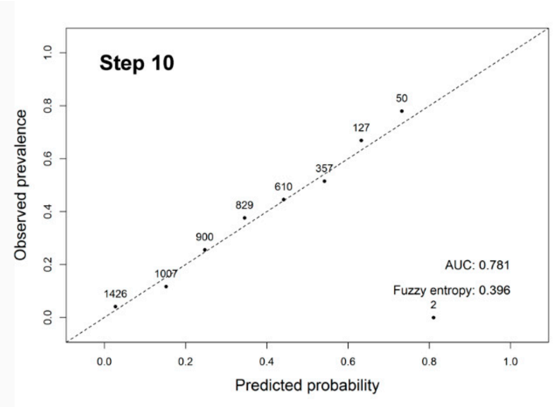

Distribution and favourability:

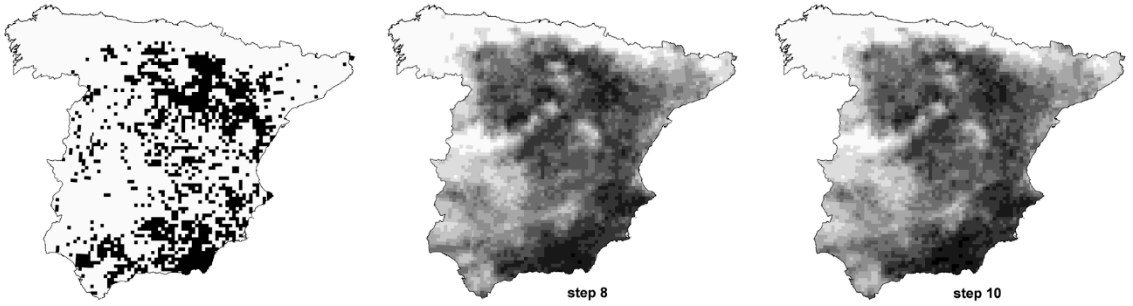

Galemys pyrenaicus

Entropy of each step:

Step	Variable	Cor_P	H	Cor_F	R	AIC
0	Intercept	-	8.58	-	1	3374.19
1	+ SIDSpr	0.816	7.60	0.917	0.204	2192.14
2	+ PET	0.918	7.46	0.933	0.162	2021.68
3	+ DAlt	0.939	7.44	0.951	0.168	1971.04
4	+ U100	0.962	7.40	0.967	0.155	1919.65
5	+ Calc	0.982	7.363	0.981	0.1472	1876.46
6	+ TRan	0.985	7.357	0.985	0.1468	1869.05
7	+ U500	0.990	7.354	0.987	0.1488	1862.62
8	+ DTn20	0.991	7.352	0.991	0.1494	1856.99
9	+ AET	0.993	7.34	0.994	0.140	1852.17
10	+ Clay	0.995	7.333	0.996	0.139	1847.81
11	+ DP30Sum	0.997	7.329	0.997	0.138	1844.66
12	+ Alt	0.9997	7.324	0.998	0.137	1841.88
13	+ Gyps	1	7.321	1	0.136	1841.69

Final favourability model (step 13):

Variables	ß	SE	Sig
Intercept	15.12	1.52	***
SIDSpr	-2.32	0.329	***
PET	-0.0195	0.00214	***
DAlt	0.00169	0.000228	***
U100	-0.0195	0.00273	***
Calc	-0.968	0.239	***
TRan	0.260	0.0571	***
U500	0.00364	0.00124	**
DTn20	0.0399	0.0138	**
AET	0.00284	0.00103	**
Clay	0.660	0.255	**
DP30Sum	-0.302	0.143	*
Alt	-0.000618	0.000274	*
Gyps	-2.10	1.75	ns

Evaluation metrics	
AUC	0.941
Kappa	0.462
Sensitivity	0.901
Specificity	0.843
CCR	0.849
HL	10.01 ns

Galemys pyrenaicus (cont.)

Calibration plot:

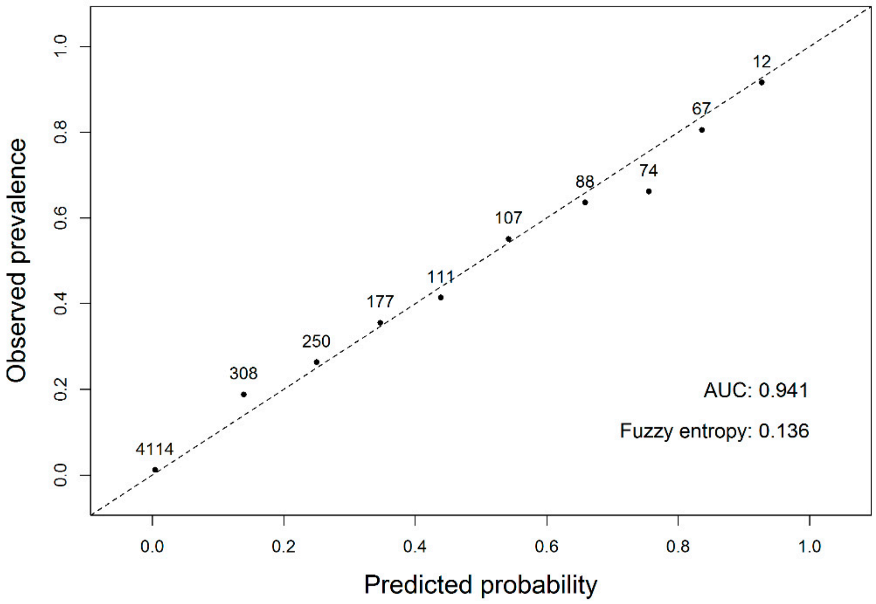

Distribution and favourability:

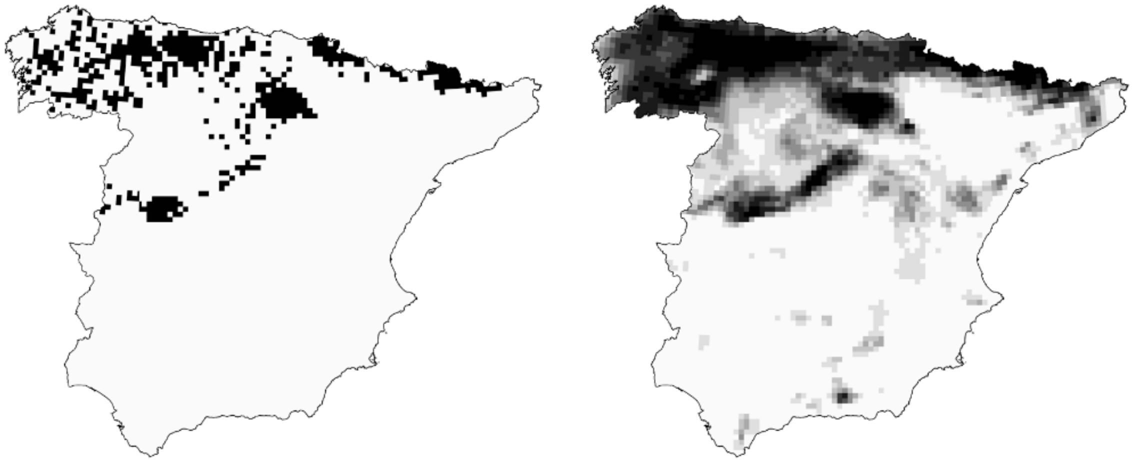

Lynx pardinus

Entropy of each step:

Step	Variable	Cor_P	H	Cor_F	R	AIC
0	Intercept	-	8.58	-	1	362.00
1	+ DTx25Aut	0.625	6.22	0.772	0.0788	224.28
2	+ SISSum	0.781	5.90	0.889	0.0603	207.11
3	+ SE	0.915	5.71	0.913	0.0548	196.38
4	+ DP30Sum	0.931	5.58	0.942	0.0471	189.76
5	+ DTn20	0.963	5.53	0.957	0.0512	188.08
6	+ U500	0.977	5.50	0.970	0.0456	187.38
7	+ ROff	0.987	5.45	0.989	0.0441	185.92
8	+ DP30Spr	1	5.41	1	0.0446	185.53

Favourability model of step 7:

Variables	ß	SE	Sig		Evaluation metrics	
Intercept	-280.80	60.19	***		AUC	0.981
DTx25Aut	0.315	0.0759	***		Kappa	0.109
SISSum	33.99	7.72	***		Sensitivity	0.897
SE	0.0665	0.0190	***		Specificity	0.927
DP30Sum	11.70	3.15	***		CCR	0.927
DTn20	-0.0554	0.0243	*		HL	4.90 ns
U500	0.0109	0.00553	*			
ROff	0.00540	0.00270	*			

Final favourability model (step 8):

Variables	ß	SE	Sig		Evaluation metrics	
Intercept	-285.80	62.51	***		AUC	0.982
DTx25Aut	0.291	0.0752	***		Kappa	0.118
SISSum	34.73	8.02	***		Sensitivity	0.931
SE	0.0685	0.0188	***		Specificity	0.930
DP30Sum	13.10	3.23	***		CCR	0.930
DTn20	-0.0445	0.0253	ns		HL	3.33 ns
U500	0.00855	0.00568	ns			
ROff	0.00888	0.00362	*			
DP30Spr	-1.22	0.822	ns			

Lynx pardinus (cont.)

Calibration plots:

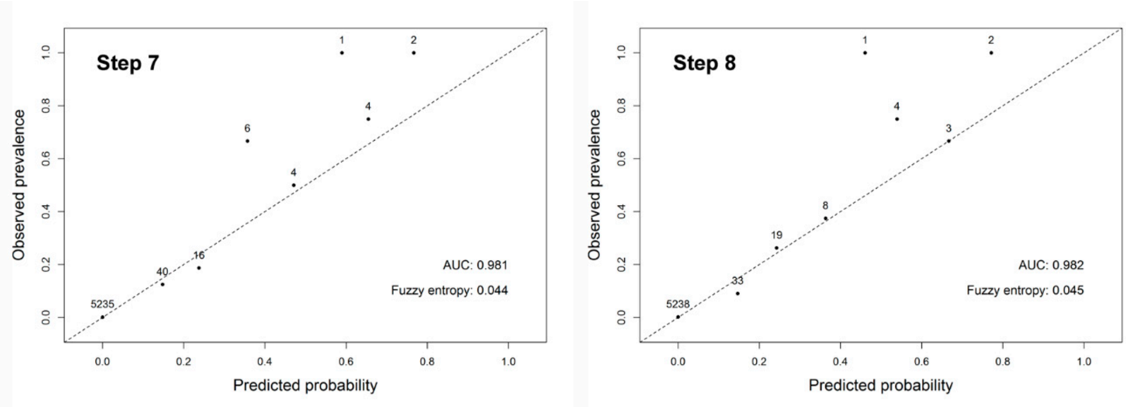

Distribution and favourability:

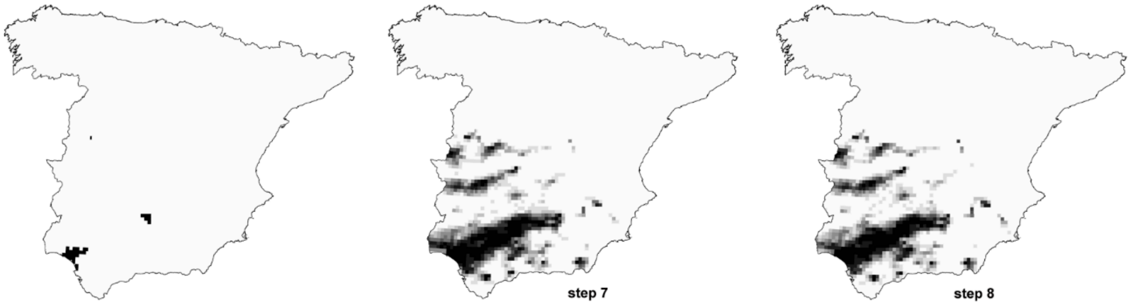

Capra pyrenaica

Entropy of each step:

Step	Variable	Cor_P	H	Cor_F	R	AIC
0	Intercept	-	8.58	-	1	3937.11
1	+ DAlt	0.464	8.35	0.466	0.572	3578.82
2	+ SISWin	0.728	8.09	0.756	0.396	3158.69
3	+ Alt	0.842	7.95	0.853	0.340	2926.03
4	+ Calc	0.906	7.86	0.898	0.296	2790.14
5	+ CTI	0.930	7.81	0.935	0.266	2717.59
6	+ Sil	0.941	7.79	0.943	0.259	2694.03
7	+ DP30Spr	0.950	7.783	0.950	0.2555	2673.58
8	+ DTn0Sum	0.958	7.776	0.955	0.2557	2659.22
9	+ WE	0.965	7.77	0.962	0.252	2643.73
10	+ U100	0.971	7.752	0.968	0.244	2629.38
11	+ Gyps	0.973	7.745	0.975	0.2402	2622.42
12	+ TnWin	0.977	7.743	0.975	0.2396	2617.60
13	+ TRan	0.982	7.736	0.983	0.239	2603.83
14	+ DP01Win	0.989	7.72	0.989	0.23147	2588.95
15	+ DTn20	0.995	7.714	0.995	0.23146	2576.05
16	+ DHi	0.997	7.710	0.997	0.2295	2571.82
17	+ Grav	0.999	7.708	0.999	0.2289	2569.47
18	+ U500	1	7.70	1	0.227	2568.37

Final favourability model (step 18):

Variables	ß	SE	Sig
Intercept	4.89	2.39	ns
DAlt	0.000381	0.000256	ns
SISWin	2.70	0.369	***
Alt	0.00335	0.000340	***
Calc	0.397	0.231	ns
CTI	-0.981	0.131	***
Sil	-1.30	0.227	***
DP30Spr	0.645	0.113	***
DTn0Sum	-0.532	0.257	*
WE	-0.0177	0.00484	***
U100	0.00510	0.00245	*
Gyps	-3.75	1.60	*
TnWin	0.419	0.0616	***
TRan	0.254	0.0466	***
DP01Win	-0.0558	0.0124	***
DTn20	-0.0202	0.00591	***
DHi	0.00793	0.00289	**
Grav	0.868	0.379	*
U500	-0.00206	0.00117	ns

Evaluation metrics	
AUC	0.896
Kappa	0.416
Sensitivity	0.838
Specificity	0.803
CCR	0.808
HL	13.10 ns

Capra pyrenaica (cont.)

Calibration plot:

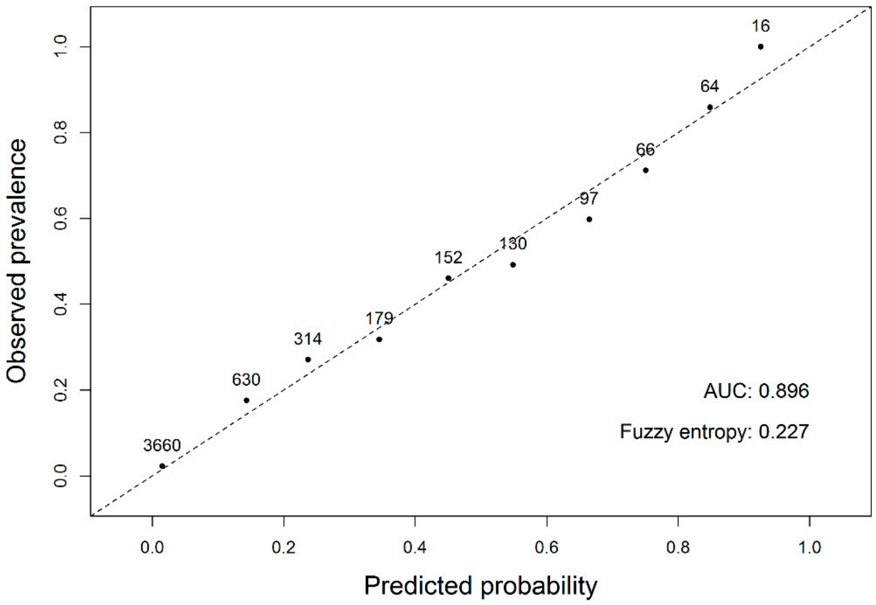

Distribution and favourability:

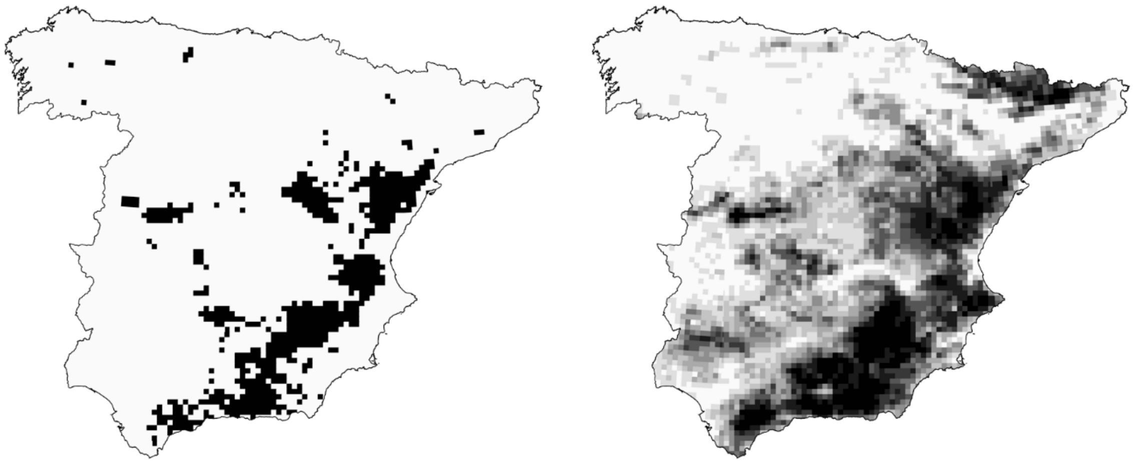

Microtus cabrerae

Entropy of each step:

Step	Variable	Cor_P	H	Cor_F	R	AIC
0	Intercept	-	8.58	-	1	2148.13
1	+ TRan	0.330	8.34	0.586	0.518	2012.46
2	+ AET	0.589	8.10	0.712	0.415	1870.47
3	+ PET	0.706	7.92	0.836	0.335	1770.52
4	+ SIDSum	0.849	7.77	0.930	0.278	1679.36
5	+ Calc	0.890	7.72	0.954	0.265	1649.60
6	+ DTn20Aut	0.924	7.68	0.969	0.251	1629.98
7	+ CTI	0.957	7.65	0.980	0.2455	1613.24
8	+ TnWin	0.980	7.63	0.990	0.2463	1599.77
9	+ U500	0.994	7.62	0.996	0.2468	1592.70
10	+ Alt	0.997	7.613	0.998	0.2471	1592.21
11	+ DP30Sum	1	7.610	1	0.2464	1592.13

Favourability model of step 7:

Variables	ß	SE	Sig	Evaluation metrics	
Intercept	-11.12	1.97	***	AUC	0.864
TRan	0.446	0.0673	***	Kappa	0.172
AET	0.00661	0.000709	***	Sensitivity	0.787
PET	-0.0145	0.00142	***	Specificity	0.748
SIDSum	3.30	0.319	***	CCR	0.750
Calc	1.12	0.126	***	HL	5.53 ns
DTn20Aut	-0.302	0.0671	***		
CTI	-0.400	0.0940	***		

Final favourability model (step 11):

Variables	ß	SE	Sig	Evaluation metrics	
Intercept	-11.08	3.11	***	AUC	0.871
TRan	0.618	0.0810	***	Kappa	0.183
AET	0.00641	0.000722	***	Sensitivity	0.805
PET	-0.0181	0.00193	***	Specificity	0.755
SIDSum	2.92	0.420	***	CCR	0.757
Calc	1.07	0.231	***	HL	7.43 ns
DTn20Aut	-0.408	0.0724	***		
CTI	-0.366	0.15	**		
TnWin	0.370	0.0822	***		
U500	0.00387	0.00133	**		
Alt	0.000830	0.000447	ns		
DP30Sum	0.443	0.307	ns		

Microtus cabrerae (cont.)

Calibration plots:

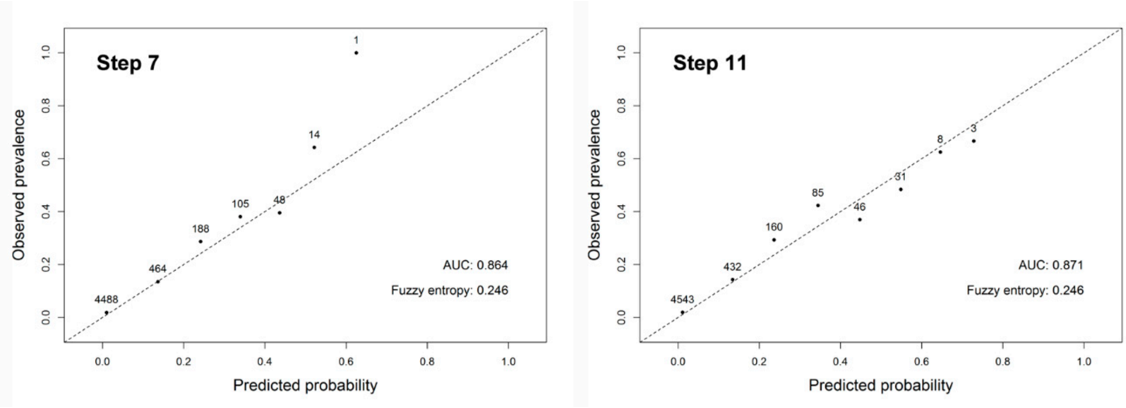

Distribution and favourability:

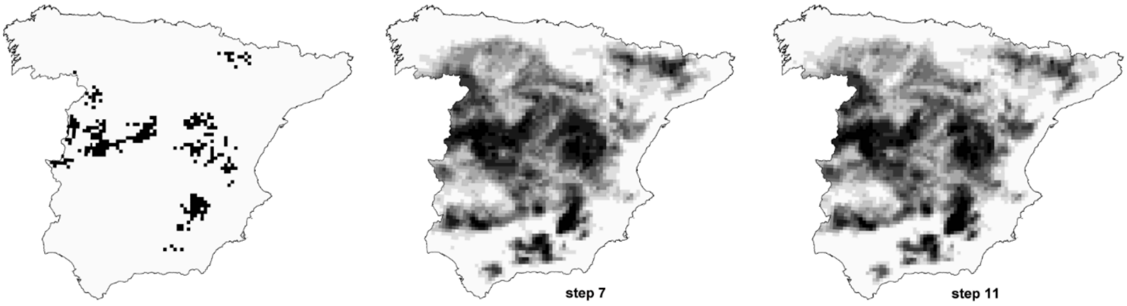

Lepus castroviejoi

Entropy of each step:

Step	Variable	Cor_P	H	Cor_F	R	AIC
0	Intercept	-	8.58	-	1	738.41
1	+ AET	0.529	6.17	0.689	0.0683	391.09
2	+ TRan	0.761	5.60	0.858	0.0354	298.52
3	+ DP30Sum	0.829	5.47	0.878	0.0297	277.41
4	+ CTI	0.908	5.25	0.954	0.0213	239.33
5	+ DP30Spr	0.948	5.17	0.960	0.0186	227.29
6	+ U500	0.961	5.14	0.974	0.0178	222.68
7	+ Alt	0.975	5.11	0.983	0.0184	217.55
8	+ Clay	0.987	5.08	0.991	0.0180	213.35
9	+ ROff	0.995	5.06	0.994	0.0182	211.26
10	+ SE	1	5.05	1	0.0187	209.94

Favourability model of step 6:

Variables	ß	SE	Sig	Evaluation metrics	
Intercept	94.78	11.63	***	AUC	0.994
AET	-0.0712	0.0108	***	Kappa	0.385
TRan	-0.656	0.149	***	Sensitivity	0.986
DP30Sum	-3.55	0.597	***	Specificity	0.961
CTI	-2.48	0.526	***	CCR	0.961
DP30Spr	1.057	0.270	***	HL	8.12 ns
U500	0.00900	0.00362	*		

Final favourability model (step 10):

Variables	ß	SE	Sig	Evaluation metrics	
Intercept	69.81	12.50	***	AUC	0.995
AET	-0.0558	0.0112	***	Kappa	0.402
TRan	-0.837	0.214	***	Sensitivity	0.986
DP30Sum	-3.779	0.744	***	Specificity	0.964
CTI	-1.557	0.608	*	CCR	0.964
DP30Spr	1.08	0.324	***	HL	4.76 ns
U500	0.0182	0.00496	***		
Alt	0.00314	0.00101	**		
Clay	2.62	1.04	*		
ROff	0.00136	0.000715	ns		
SE	-0.0398	0.0218	ns		

Lepus castroviejoi (cont.)

Calibration plots:

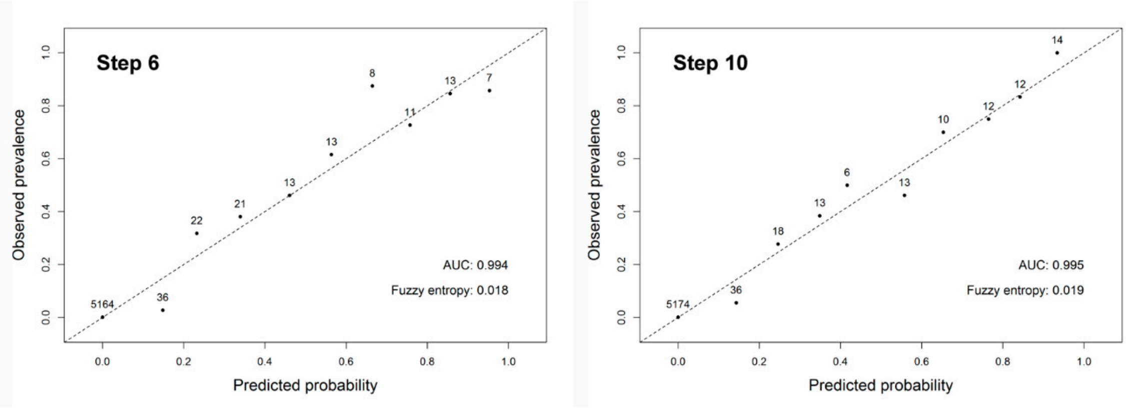

Distribution and favourability:

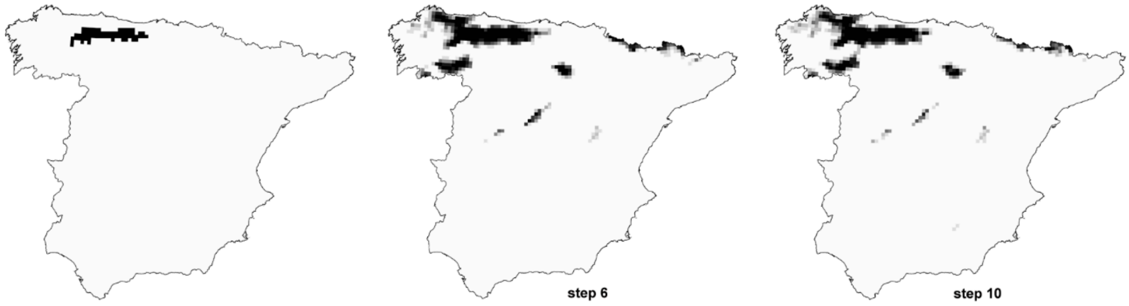

Supplement: Supplementary file 1 [file entropy-23-01014-s001.zip › Supplementary Materials S1.pdf]
