# Supplementary material for: A Stepwise Assessment of Parsimony and Fuzzy Entropy in Species Distribution Modelling
Source: Entropy (Basel). 2021 Aug 5;23(8):1014. doi: 10.3390/e23081014 (PMC8392680; doi:10.3390/e23081014)
Supplement: Supplementary file 1 [file entropy-23-01014-s001.zip › Supplementary Materials S2.pdf]

A stepwise assessment of parsimony and fuzzy entropy in species distribution modelling

Alba Estrada & Raimundo Real

Supplementary Materials S2.

Calibration plots, AUC values and fuzzy entropy of each step of the models of *Chalcides bedriagai* and *Sylvia conspicillata* (those studied species with lower values of AUC). Numbers in the calibration plots indicate the sample size of each bin.

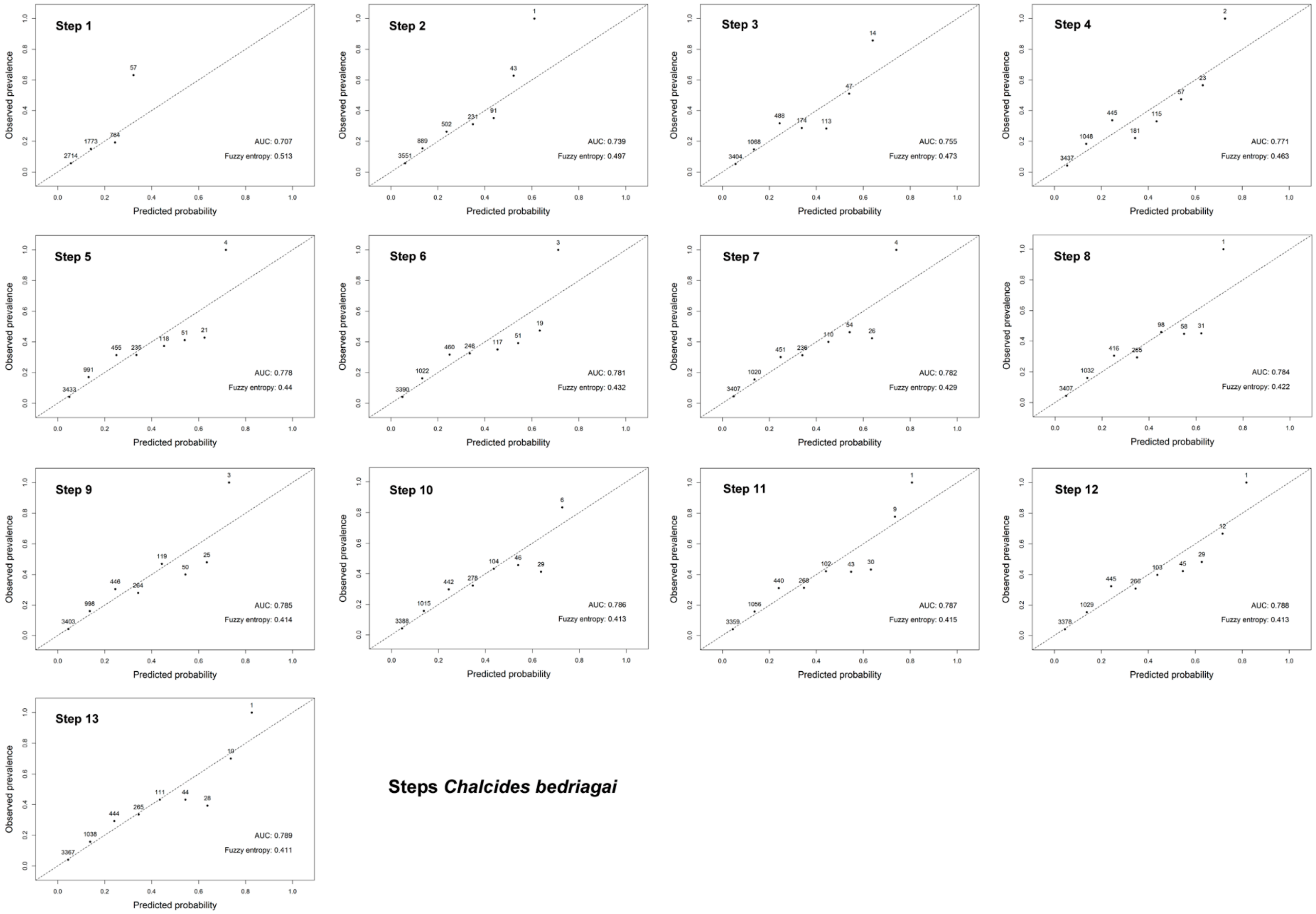

Steps *Chalcides bedriagai*

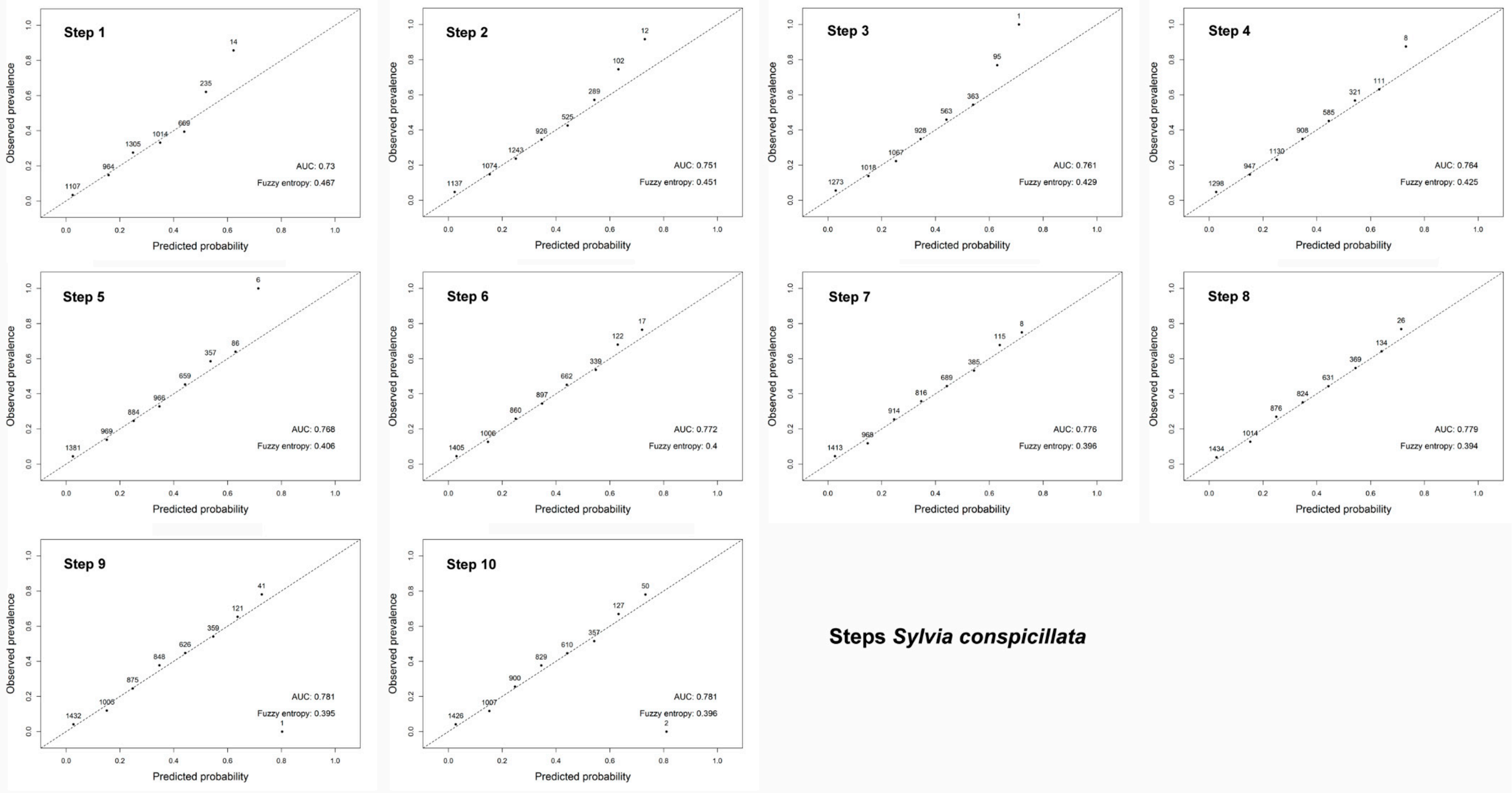

Steps *Sylvia conspicillata*
