# Supplementary material for: A Stepwise Assessment of Parsimony and Fuzzy Entropy in Species Distribution Modelling
Source: Entropy (Basel). 2021 Aug 5;23(8):1014. doi: 10.3390/e23081014 (PMC8392680; doi:10.3390/e23081014)
Supplement: Supplementary file 1 [file entropy-23-01014-s001.zip › Table S1.pdf]

A stepwise assessment of parsimony and fuzzy entropy in species distribution modelling

Alba Estrada & Raimundo Real

Table S1. Environmental variables considered. In all cases, variables are the average value in the UTM cell. For each species, we performed a selection of variables in three stages (see Methods).

Variable	Code	Variable	Code
**Climate**		**Climate**	
Mean annual temperature (ºC)	Tann[1]	Total precipitation in summer	PSum
Mean temperature in spring	TSpr	Total precipitation in autumn	PAut
Mean temperature in summer	TSum	Total precipitation in winter	PWin
Mean temperature in autumn	TAut	Days with precipitation ≥0.1mm	DP01
Mean temperature in winter	TWin	Days with precipitation ≥0.1mm in spring	DP01Spr
Mean temperature in January	TJan	Days with precipitation ≥0.1mm in summer	DP01Sum
Mean temperature in July	TJul	Days with precipitation ≥0.1mm in autumn	DP01Aut
Annual temperature range (=TJul - TJan)	TRan	Days with precipitation ≥0.1mm in winter	DP01Win
Minimum annual temperature	TnAnn	Days with precipitation ≥1mm	DP1
Minimum temperature in spring	TnSpr	Days with precipitation ≥1mm in spring	DP1Spr
Minimum temperature in summer	TnSum	Days with precipitation ≥1mm in summer	DP1Sum
Minimum temperature in autumn	TnAut	Days with precipitation ≥1mm in autumn	DP1Aut
Minimum temperature in winter	TnWin	Days with precipitation ≥1mm in winter	DP1Win
Minimum temperature in January	TnJan	Days with precipitation ≥10mm	DP10
Minimum temperature in July	TnJul	Days with precipitation ≥10mm in spring	DP10Spr
Maximum annual temperature	TxAnn	Days with precipitation ≥10mm in summer	DP10Sum
Maximum temperature in spring	TxSpr	Days with precipitation ≥10mm in autumn	DP10Aut
Maximum temperature in summer	TxSum	Days with precipitation ≥10mm in winter	DP10Win
Maximum temperature in autumn	TxAut	Days with precipitation ≥30mm	DP30
Maximum temperature in winter	TxWin	Days with precipitation ≥30mm in spring	DP30Spr
Maximum temperature in January	TxJan	Days with precipitation ≥30mm in summer	DP30Sum
Maximum temperature in July	TxJul	Days with precipitation ≥30mm in autumn	DP30Aut
Direct Irradiance at Surface ($kWh/m^2/d$)	SID[2]	Days with precipitation ≥30mm in winter	DP30Win
Direct Irradiance at Surface in spring	SIDSpr	Mean annual runoff (mm)	ROff[3]
Direct Irradiance at Surface in summer	SIDSum	Mean annual potential evapotranspiration (mm)	PET[4]
Direct Irradiance at Surface in autumn	SIDAut	Mean annual actual evapotranspiration (mm) (=min [PAnn, PET])	AET
Direct Irradiance at Surface in winter	SIDWin	**Topography**	
Surface Incoming Radiation ($kWh/m^2/d$)	SIS	Mean altitude (m)	Alt[5]
Surface Incoming Radiation in spring	SISSpr	Altitude difference (calculated from Alt)	DAlt
Surface Incoming Radiation in summer	SISSum	Slope (º) (calculated from Alt)	Slop
Surface Incoming Radiation in autumn	SISAut	Southward exposition degree	SE[6]

Surface Incoming Radiation in winter	SISWin	Westward exposition degree	WE[6]
Days with minimum temperature ≤0ºC	DTn0	Hydrologically conditioned compound topographic index	CTI[7]
Days with minimum temperature ≤0ºC in spring	DTn0Spr	**Lithology**	
Days with minimum temperature ≤0ºC in summer	DTn0Sum	Proportion of clay	Clay[8]
Days with minimum temperature ≤0ºC in autumn	DTn0Aut	Proportion of silica	Sil
Days with minimum temperature ≤0ºC in winter	DTn0Win	Proportion of calcareous rock	Calc
Days with minimum temperature ≥20ºC	DTn20	Proportion of gravels	Grav
Days with minimum temperature ≥20ºC in autumn	DTn20Aut	Proportion of gypsum	Gyps
Days with maximum temperature ≥25ºC	DTx25	**Human activity**	
Days with maximum temperature ≥25ºC in spring	DTx25Spr	Distance to the nearest highway (km)	DHi[9]
Days with maximum temperature ≥25ºC in summer	DTx25Sum	Distance to the nearest urban centre with more than 100,000 inhabitants (km)	U100
Days with maximum temperature ≥25ºC in autumn	DTx25Aut	Distance to the nearest urban centre with more than 500,000 inhabitants (km)	U500
Total annual precipitation (mm)	PAnn	Human population density (number of inhabitants/km^2)	HPd[10]
Total precipitation in spring	PSpr		

Sources: 1: from *Tann* to *TxJul* and from *DTn0* to *DP30Win*: Agencia Estatal de Meteorología (2011); 2: from *SID* to *SISWin*: Sancho *et al.* (2012); 3: IGME (1979); 4: Font (2000); 5: US Geological Survey (1996); 6: Farr and Kobrick (2000); 7: http://hydrosheds.cr.usgs.gov; 8: from *Clay* to *Gyps*: OneGeology-Europe (2015); 9: from *DHi* to *U500*: IGN (1999); 10: ORNL (2001).

References

Agencia Estatal de Meteorología (2011) *Iberian climate atlas. Air temperature and precipitation (1971-2000)*. Agencia Estatal de Meteorología. Ministerio de Medio Ambiente y Medio Rural y Marino.

Farr, T.G. & Kobrick, M. (2000) Shuttle radar topography mission produces a wealth of data. *Eos Trans. Am. Geophys. Union*, **81**, 583–585.

Font, I. (2000) *Climatología de España y Portugal*. Ediciones Universidad de Salamanca.

IGME (1979) *Mapa hidrogeológico nacional. Explicación de los mapas de lluvia útil, de reconocimiento hidrogeológico y de síntesis de los sistemas acuíferos. Vol. 81, 2ª ed.* Instituto Geológico y Minero de España. Ministerio de Industria y Energía, Madrid.

IGN (1999) *Mapa de carreteras. Península Ibérica, Baleares y Canarias*. Instituto Geográfico Nacional, Ministerio de Fomento, Madrid.

OneGeology-Europe (2015) http://www.onegeology-europe.org/.

ORNL (2001) *LandScan 2000 Global Population Database*. Oak Ridge National Laboratory (ORNL), Oak Ridge, TN.

Sancho, J.M., Riesco, J., Jiménez, C., Sánchez de Cos, M.C., Montero, J. & López, M. (2012) *Atlas de radiación solar en España utilizando datos del SAF de clima de EUMETSAT*. Agencia Estatal de Meteorología. Ministerio de Agricultura, Alimentación y Medio Ambiente.

US Geological Survey (1996) GTOPO30. Land Processes Distributed Active Archive Center. <http://www.edcdaac.usgs.gov/gtopo30/gtopo30.asp>.
